# Genome-wide landscape of genetic diversity, runs of homozygosity, and runs of heterozygosity in five Alpine and Mediterranean goat breeds

**DOI:** 10.1186/s40104-025-01155-3

**Published:** 2025-03-03

**Authors:** Sara Pegolo, Vittoria Bisutti, Lucio Flavio Macedo Mota, Alessio Cecchinato, Nicolò Amalfitano, Maria Luisa Dettori, Michele Pazzola, Giuseppe Massimo Vacca, Giovanni Bittante

**Affiliations:** 1 Department of Agronomy, Food, Natural Resources, Animals and Environment (DAFNAE), University of Padova (Padua), Viale Dell’Università 16, 35020 Legnaro, PD Italy; 2 Department of Veterinary Medicine, University of Sassari, Via Vienna 2, 07100 Sassari, SS Italy

**Keywords:** Candidate genes, Genomic diversity, Resilience, ROH, ROHet, Sustainability

## Abstract

**Background:**

Goat breeds in the Alpine area and Mediterranean basin exhibit a unique genetic heritage shaped by centuries of selection and adaptability to harsh environments. Understanding their adaptive traits can aid breeding programs target enhanced resilience and productivity, especially as we are facing important climate and agriculture challenges. To this aim the genomic architecture of 480 goats belonging to five breeds (i.e., Saanen [SAA], Camosciata delle Alpi [CAM], Murciano-Granadina [MUR], Maltese [MAL], Sarda [SAR]) reared in the Sardinia Island were genotyped and their genomic architecture evaluated to find molecular basis of adaptive traits. Inbreeding, runs of homozygosity (ROH) and runs of heterozygosity (ROHet) were identified. Finally, candidate genes in the ROH and ROHet regions were explored through a pathway analysis to assess their molecular role.

**Results:**

In total, we detected 10,341 ROH in the SAA genome, 11,063 ROH in the CAM genome, 12,250 ROH in the MUR genome, 8,939 ROH in the MAL genome, and 18,441 ROH in the SAR genome. Moreover, we identified 4,087 ROHet for SAA, 3,360 for CAM, 2,927 for MUR, 3,701 for MAL, and 3,576 for SAR, with SAR having the highest heterozygosity coefficient. Interestingly, when computing the inbreeding coefficient using homozygous segment (*F*_ROH_), SAA showed the lowest value while MAL the highest one, suggesting the need to improve selecting strategies to preserve genetic diversity within the population. Among the most significant candidate genes, we identified several ones linked to different physiological functions, such as milk production (e.g., *DGAT1*, *B4GALT1*), immunity (*GABARAP*, *GPS2*) and adaptation to environment (e.g., *GJA3*, *GJB2* and *GJB6*).

**Conclusions:**

This study highlighted the genetic diversity within and among five goat breeds. The high levels of ROH identified in some breeds might indicate high levels of inbreeding and a lack in genetic variation, which might negatively impact the animal population. Conversely, high levels of ROHet might indicate regions of the genetic diversity, beneficial for breed health and resilience. Therefore, these findings could aid breeding programs in managing inbreeding and preserving genetic diversity.

**Supplementary Information:**

The online version contains supplementary material available at 10.1186/s40104-025-01155-3.

## Background

Local goat breeds endemic to various areas of the Mediterranean basin represent a reservoir of genetic diversity crucial for biodiversity conservation and sustainable agroecosystems. These indigenous breeds, shaped by centuries of natural selection and environmental pressures, exhibit phenotypic and genotypic adaptations that make them well-suited to areas otherwise not appropriate for intensive livestock farming [1]. However, these local goat breeds are increasingly experiencing a process of partial farming intensification, coupled with the introduction of more productive breeds from very different areas, especially those of Alpine origin. Their genetic makeup reflects a mosaic of evolutionary processes, including genetic drift, founder effects, and local adaptation, encapsulating a unique genetic heritage.

In diploid genomes, runs of homozygosity (ROH) represent uninterrupted stretches of homozygous DNA sequences [2]. These ROH formations are predominantly influenced by demographic events such as population bottlenecks, genetic drift, and inbreeding [3]. These continuous DNA segments play a pivotal role in assessing levels of inbreeding within livestock species: longer ROH segments signify recent inbreeding events, while shorter segments indicate inbreeding that occurred in earlier generations [4]. The distribution of ROH fragments within chromosomes exhibits a nonrandom pattern, with numerous molecular markers displaying atypical frequencies within ROH, referred to as "ROH hotspots" [5]. A growing body of research has substantiated that these ROH hotspots are subject to positive selection across various livestock species, including goats [6, 7]. Positive selection typically amplifies homozygosity within the specific region of interest [8] and generally facilitates the dissemination of advantageous alleles while eliminating detrimental ones [9]. Nonetheless, numerous deleterious mutations, co-occurring in linkage disequilibrium with advantageous mutations, also experience an uptick in frequency due to allele surfing [10].

Examining ROH islands represents a highly efficient method for pinpointing genomic regions subjected to selective pressures, as these regions may harbor variants shared among individuals within a specific population [11]. Few studies have investigated the pattern of ROH in goat breeds [6, 12].

Runs of heterozygosity (ROHet) are stretches of the genome in an individual where consecutive markers (usually single nucleotide polymorphisms, or SNPs) carry heterozygous alleles [13]. These stretches can vary in length, ranging from just a few consecutive markers to long segments encompassing multiple genes. The detection of genomic regions with high genetic variability offers insights into the levels of population genetic diversity and evolutionary history. Furthermore, it enables the pinpointing of particular genome segments where preserving higher genetic diversity could yield significant benefits [14]. Previous studies identified ROHet hotspots in various livestock species [13–15], including goats [16, 17]. However, despite these findings, this topic still requires further extensive investigation.

In the face of climate change and emerging agricultural challenges, the adaptive traits encoded within local breed goats in Mediterranean areas offer valuable resources for breeding programs aimed at enhancing resilience and productivity. Sardinia, a region of Italy and the second largest island of the Mediterranean Sea, strongly relies on agriculture and livestock farming as crucial sectors of its local economic income [18]. Since the Neolithic age, Sardinia has served as a key stopover of Mediterranean Sea routes connecting African and European territories [19]. The transportation of animals from and to Sardinia, and the resultant gene flow, is reported for many species, such as pigs, cattle, and goats [20]. Goats, in particular, are normally allowed to pasture in extensive lands, with consequent interactions and gene flow among the different herds [1]. A large research project based on Sardinia Island enabled us to study and compare several goat breeds of Mediterranean and Alpine origin in an area characterized by the presence of different farming systems, from the very extensive to the intensive ones. These previous studies allowed us to characterize goat breeds and farming systems in relation to quantitative and qualitative production of milk [21–23] as well as their cheese-making ability [24–26].

In this context, Sardinian goat farming, including its systems, breeds, and crossings, represents a valuable case study. Indeed, some goat breeds are multi-purpose autochthonous, while others are specialized imported breeds. The Sarda is the Sardinian autochthonous breed, exclusively managed in semi-extensive farms which are mostly located in mountainous areas. The Camosciata delle Alpi, the Italian goat breed corresponding to the Alpine Chamois, and the Saanen are two dairy-specialized breeds of Swiss origins which were introduced to Sardinia to be initially farmed in mixed systems in the mountains, and later in intensive ones located in the plains. The Maltese is a Mediterranean breed from Sicily (Italy), which has been widely crossed since the 80s with the local Sarda breed to improve milk yield; it has now nearly disappeared from Sardinian farms, replaced by other specialized breeds. Finally, the Murciano-Granadina, a breed from Spain, has been recently introduced in mono-breed intensive farms located in the plains [18].

By elucidating the genomic architecture and functional significance of genetic variants in goat populations reared on Sardinia Island, we aim to uncover the molecular basis of adaptive traits and develop conservation strategies to safeguard these genetic resources. Therefore, we employed in this study the GGP 70k chip available for goats to characterize ROH and ROHet in 480 goats from five different Alpine and Mediterranean breeds reared in Sardinia and estimate their inbreeding level as a contribution to biodiversity preservation and prioritization strategies.

## Methods

### Animals and sampling

All procedures were approved by the Ethical Animal Care and Experimental Use Committee (Organismo Preposto al Benessere e alla Sperimentazione Animale, OPBSA) at the University of Sassari (protocol number 0122930, approved on 28 September 2021). The 480 sampled goats were all lactating females reared in 35 different farms located in the territory of Sardinia (Fig. S1). Goats were all registered in the official herd book of the following breeds:

- Saanen (SAA), *n* = 97 goats in 10 farms, from 3 to 16 sampled goats per farm;
- Camosciata delle Alpi (CAM), *n* = 88 goats in 7 farms, from a minimum of 8 to a maximum of 16 sampled goats per farm;
- Murciano-Granadina (MUR), *n* = 87 goats in 8 farms, from 2 to 16 sampled goats per farm;
- Maltese (MAL), *n* = 96 goats in 7 farms, from 8 to 17 sampled goats per farm;
- Sarda (SAR), *n* = 112 goats in 11 farms, from 10 to 16 sampled goats per farm.

Details of animals, breeds and farms are reported in Vacca et al. [25].

Individual blood samples were collected from the jugular vein in K3EDTA vacuum tubes (Vacutest Kima, Azergrande, PD, Italy), and DNA was later extracted at the laboratories using a commercial kit (Gentra Puregene Blood Kit, QUIAGEN, Venlo, The Netherlands), following the manufacturer’s instruction.

### Genomic information

The genomic data file was generated from 502 animals genotyped with 70,000 SNP markers using the Geneseek Genomic Profiler Goat 70K chip (Neogen Europe Ltd, Auchincruive, UK). The quality control (QC) of genomic information was performed by removing autosomal markers with a GenCall score lower than 0.6 to remove genotyping error, minor allele frequency (MAF) lower than 0.05, a significant deviation from Hardy–Weinberg equilibrium (*P* ≤ 10^–5^), and a call rate of markers and samples lower than 0.90. After quality control, a total of 480 animals and 51,940 SNP markers have remained for estimates of the genomic relationship among animals. For the analyses of ROH and ROHet, we pruned autosomal markers based on a GenCall score of lower than 0.60 and a call rate of lower than 0.90, and 63,523 markers remaining for further analysis.

### Phylogenetic and principal components analysis

The phylogenetic tree was assessed using the genetic distances between animals of the different goat breeds, which were calculated based on the SNP markers information using the Plink 2.0 software [27], using the genomic information with the quality control performed. Using this distance matrix, a Neighbor-joining tree was created with R studio (R v.4.4.1, R studio v 2024.04.2) using the ape R package (v. 5.8) [28]. Principal component analysis (PCA) evaluated the population substructure based on the SNP markers using the ade4 R package (v.1.7) [29].

### Identification of runs of homozygosity (ROH) and runs of heterozygosity (ROHet)

The ROH was identified using the Plink 2.0 software [27], for the 480 animals genotyped with 77K SNP considering the following criteria: 1) a minimum of 50 SNPs in homozygosity (*–homozyg-window-snp*), 2) density of 1 SNP per 100 kb (*–homozyg-density*), 3) a maximum value of 500 kb for the gap between two consecutive SNP markers (*–homozyg-gap*), 4) 1 heterozygous locus in the ROH segment (*–homozyg-window-het*), 5) homozygous length greater than 1 Mb (–homozyg-kb), and 6) no more than 1 missing genotypes across the animals (*–homozyg window-missing*). The sum of ROH segments per goat was calculated considering four classes in megabases (Mb): between 1 and 2 Mb, 2 and 4 Mb, 4 and 8 Mb and higher than 8 Mb.

The estimation of ROHet was performed using consecutive heterozygous SNP markers using the R package detectRUNS2 (v. 0.9.6) [30]. The ROHet was performed using the following criteria [14–16]: i) the inclusion of a minimum of 15 consecutive SNPs in a ROHet, ii) a minimum length of 500 kb for a ROHet, and iii) the allowance of a maximum of two SNPs with missing genotypes and a maximum of three homozygous genotypes in a ROHet. The length of the ROHet for each animal was classified into four categories, 0.5–1 Mb, 1–1.5 Mb, 1.5–2 Mb, and > 2 Mb [14], and the resulting number and percentage of ROHet in each category were computed.

### Estimation of inbreeding coefficients

#### ***Inbreeding coefficient using homozygous segments***

The inbreeding based in ROH segments (FROH) for each animal was calculated using the following equation:

$$F_\text{ROH}=\frac{\sum_{j=1}^nL_{ROHj}}{L_{TOTAL}},$$

Where ${L}_{ROHj}$ represents the length of the ROH segment identified for each animal in bases pair (bp), *n* is the total number of ROH segments detected and ${L}_{TOTAL}$ is the total length of autosomal chromosomes covered by SNP markers in bp, which was 2,468,749,207 bp. The *F*_ROH_ also was estimated considering four classes, according to their length in megabases: between 1 and 2 Mb (*F*_ROH1_ ≤ 2 Mb), 2 and 4 Mb (*F*_ROH2_ ≤ 4 Mb), 4 and 8 Mb (*F*_ROH4_ ≤ 8 Mb) and higher than 8 Mb (*F*_ROH_ > 8 Mb).

#### Inbreeding coefficient using the genomic relationship matrix

The inbreeding estimate based on the genomic relationship matrix (*F*_G_) was obtained using the method described by VanRaden [31] using the preGSf90 program from the BLUPF90 family [32]. The genomic matrix was estimated as $G={ZZ}^{\prime}/2\sum p(1-p)$, where $Z$ is the SNP marker matrix assuming 0, 1, and 2 for genotypes AA, AB, and BB, respectively. The ${F}_{GRM}$ was estimated as the diagonal of the G matrix (${G}_{ii}$) minus 1 (${F}_{GRM}=\boldsymbol{ }{G}_{ii}-1$).

#### Inbreeding coefficient using excess homozygosity $\left(F_{exH}\right)$

The ${F}_\text{exH}$ was estimated based on the excess of homozygosity in autosomal chromosome for each animal as follows:

$$F_{exH}=\frac{Obs_{homo}-Exp_{homo}}{Total_{observation}-Exp_{homo}},$$

where ${Obs}_{homo}$ and ${Exp}_{homo}$ represents the observed and expected number of homozygous genotypes in the population, respectively. The ${F}_{exH}$ was calculated using PLINK program v. 2.0 [27].

Pearson’s correlation coefficient was used to evaluate the relationship between various estimated inbreeding coefficients, incorporating data from all animals with available inbreeding estimates.

### Detection of ROH and ROHet Hotspots

The estimation of the proportion of homozygosity per site was accomplished by determining the ratio of animals exhibiting homozygous genotypes for a specific SNP to the total number of animals genotyped for that SNP. The identification of regions in the genome with a high prevalence of ROH, commonly referred to as ROH hotspots, involved measuring the proportion of animals harboring a specific SNP within a ROH relative to the total number of animals genotyped for that SNP. Regions where at least 50% of the population exhibited ROH were classified as ROH hotspots. Overlapping segments were identified by comparing highly homozygous regions and ROH hotspots. Heterozygosity islands (ROHet) of each goat breed were determined to identify genomic regions with a high frequency of ROHet (ROHet hotspots). For this, we considered that the genomic regions in ROHet at the top 0.1% were deemed as ROHet hotspots [14, 16, 31].

### Candidate gene, pathway, and functional analyses

Candidate genes in the ROH and ROHet window regions were annotated using the Ensemble database (ARS1, http://www.ensemble.org/), considering the *Capra hircus* ARS1 assembly as a reference. Gene Ontology (GO) terms and Kyoto Encyclopedia of Genes and Genomes (KEGG) pathways were explored for functional enrichments of the candidate genes using clusterProfiler (v. 4. 4.12.2) [34] and ReactomePA (v. 1.28.0) [35], considering the Capra hircus database.

## Results

### Genetic diversity and breed relationships in goats

**Fig. 1:**
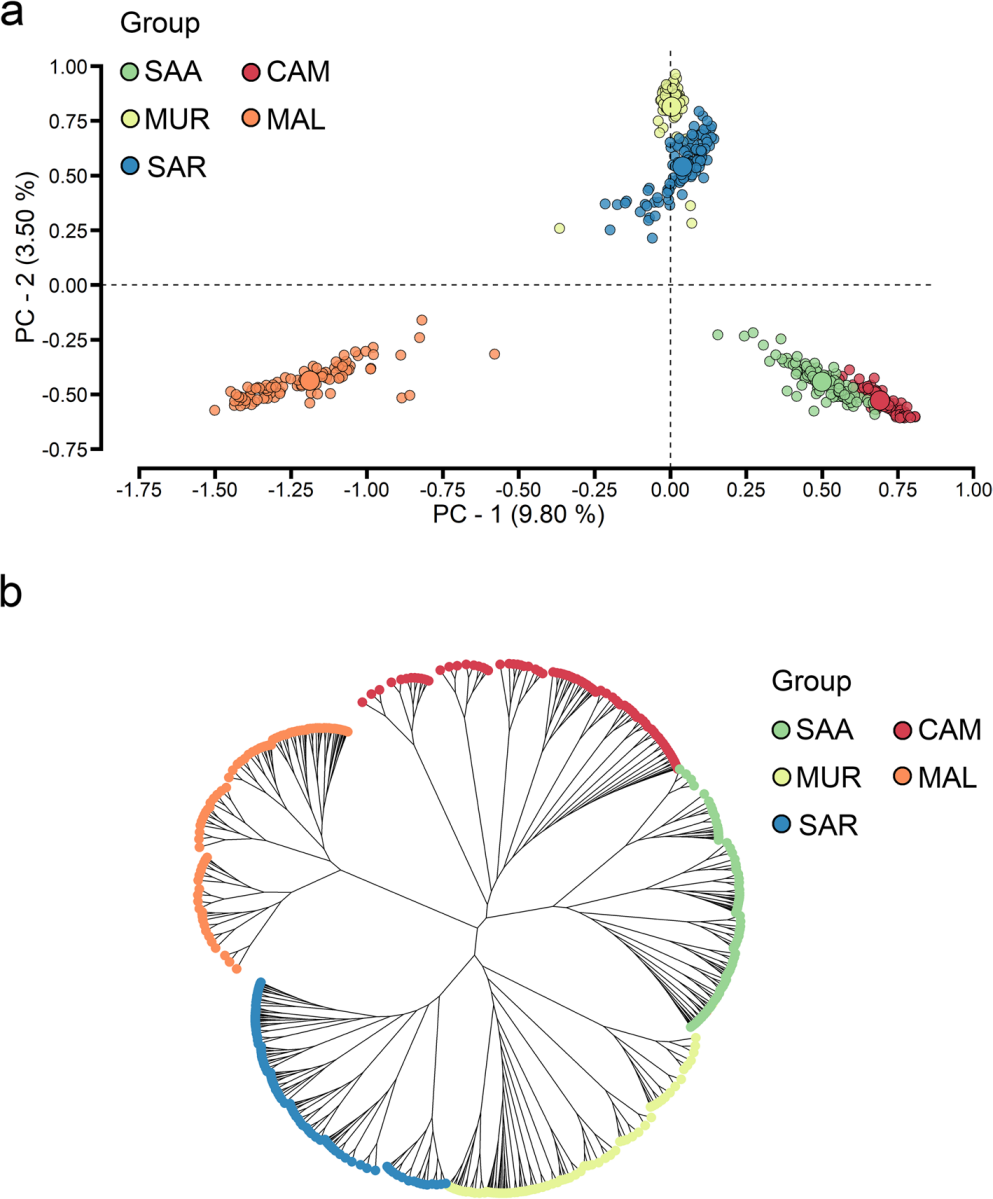
Assessment of genetic diversity among goat breeds. Principal components using genomic information (**a**) and Neighbor-joining phylogenetic tree based on genomic information (**b**). SAA: Saanen; CAM: Camosciata delle Alpi; MUR: Murciano-Granadina; MAL: Maltese; SAR: Sarda

The PC analysis of the IBS matrix derived from SNP data revealed genetic similarities between SAR and MUR, as well as between the SAA and CAM breeds, while MAL clustered separately with no overlap with the others (Fig. 1a). Specifically, the first principal component, which accounted for 9.80% of the variance, distinguished MAL from all other goat breeds. Meanwhile, the second principal component, explaining 3.50% of the variance, differentiated SAR and MUR from MAL, SAA, and CAM. These findings were further supported by the neighbor-joining phylogenetic tree, showing MAL as a distinct group, with SAR and MUR clustering together, and CAM and SAA forming another cluster (Fig. 1b). Breeds that shared close genetic relationships were placed on different branches that originated from the same basal node, i.e., CAM and SAA, MUR, and SAR breeds. In contrast, MAL was confirmed to be a more phylogenetically distant breed.

### Genomic distribution of runs of homozygosity and runs of heterozygosity

Genetic variability, measured as the amount of observed and expected heterozygosity, is reported in Table S1. The observed heterozygosity was slightly lower than the expected heterozygosity, indicating no departure from Hardy–Weinberg equilibrium (HWE) or high inbreeding levels. The percentage of polymorphic SNP was > 99%.

#### Runs of homozygosity

**Fig. 2:**
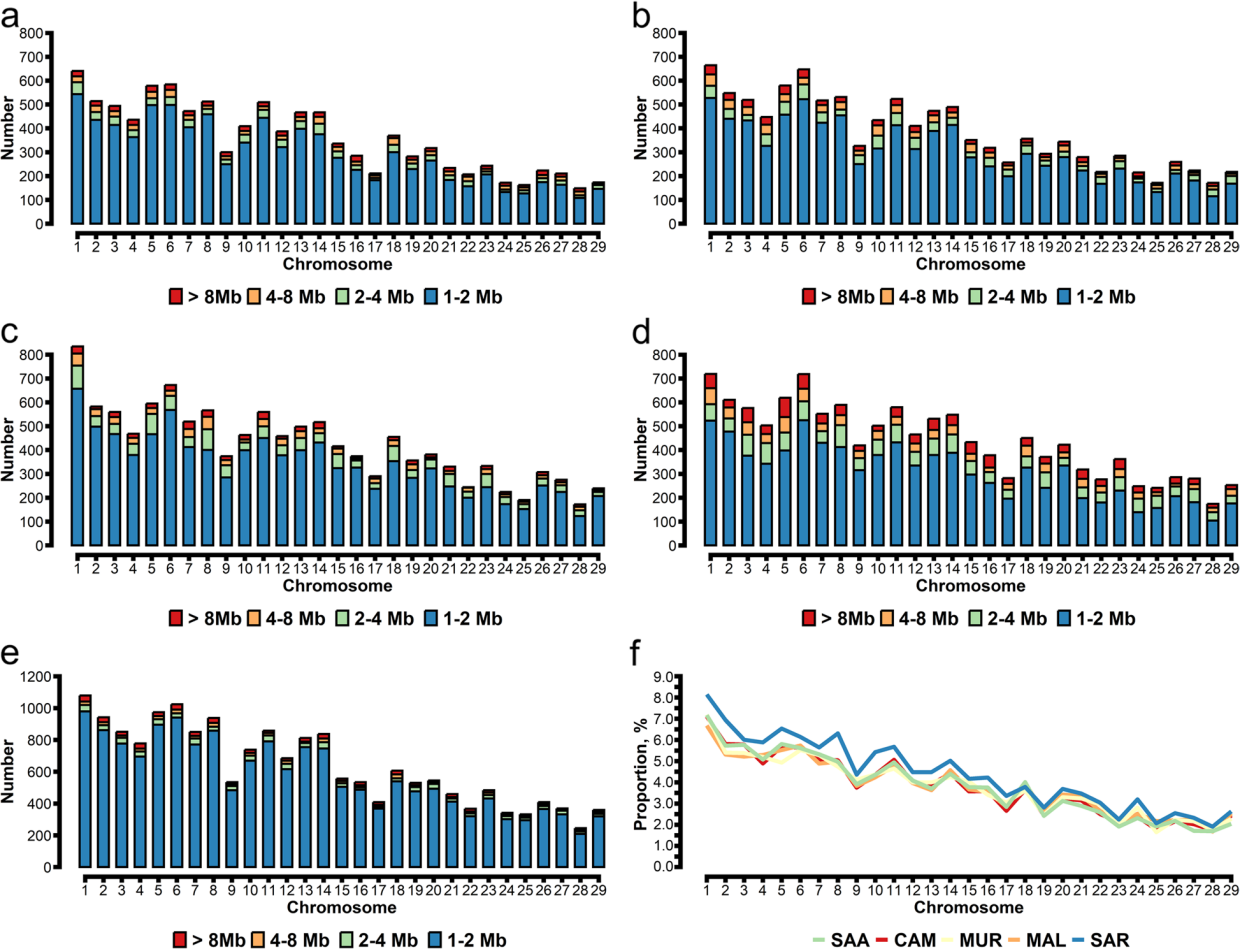
Runs of homozygosity (ROH) distribution across the genome for the different goat breeds. Runs of homozygosity (ROH) distribution for each chromosome considering different ROH length classes for different goat breeds. SAA: Saanen (**a**); CAM: Camosciata delle Alpi (**b**); MUR: Murciano-Granadina (**c**); MAL: Maltese (**d**); SAR: Sarda (**e**); Average percentage of autosome chromosome covered by ROH in goats (**f**)

In total, we detected 10,341 ROH in the SAA genome, 11,063 ROH in the CAM genome, 12,250 ROH in the MUR genome, 8,939 ROH in the MAL genome, and 18,441 ROH in the SAR genome (Table 1). The average number of ROH segments was 107.72 for SAA, 125.72 for CAM, 140.8 for MUR, 132.34 for MAL, and 164.65 for SAR (Table S2). Regarding ROH length distribution, we found a higher proportion of 1–2 Mb segments for all breeds, ranging from 70.36% of homozygosity coverage for MAL to 90.50% for SAR. In terms of specific ROH length categories, SAR exhibited the highest count of 1–2 Mb segments compared to other breeds (*P* < 0.05). MAL demonstrates the highest count of 2–4 Mb segments compared to other breeds, with MUR surpassing CAM, SAA, and SAR (*P* < 0.05). MAL also showed the highest count of 4–8 Mb segments compared to other breeds, with CAM and MUR surpassing SAR (*P* < 0.05). Furthermore, MAL displayed the highest count of 8 Mb segments compared to other breeds (*P* < 0.05; Table 1). When considering the average ROH length, SAR had the lowest value (213.12 Mb), and MAL confirmed to have the highest value (295.70 Mb) (Table S2). The ROH distribution for each breed across the autosomes is displayed in Fig. 2. The highest numbers of ROH were detected on CHR 1 and CHR 6 for CAM (664 and 647), MAL (718 and 719), and MUR (834 and 672), while a high occurrence of ROH on CHR 1 and CHR 5 was observed for SAA (640 and 580) and SAR (1,079 and 1,024) (Fig. 2a–f). The ratio of the ROH length on each autosome was calculated, and the results showed that the SAR breed had higher ratios than the other breeds across 29 autosomes. The differences ranged from 0.09% (CHR 23) to 1.27% (CHR 8) compared with CAM; from −0.19% (CHR 18) to 1.63% (CHR 2) compared with MAL; from 0.08% (CHR 22) to 1.61% (CHR 5) compared with MUR; from −0.23% (CHR 18) to 1.34% (CHR 8) compared with SAA (Fig. 2f).

**Table 1:** Descriptive statistics of runs of homozygosity (ROH) number and coverage across the genome for different length classes in mega basis (Mb)

Item	Alpine breeds		Mediterranean breeds		
	SAA	CAM	MUR	MAL	SAR
Homozygosity number					
ROH 1–2 Mb	8,616^b^(12)	8,809^b^(14)	9,856^b^(14)	8,939^b^(11)	16,690^a^(26)
ROH 2–4 Mb	725^c^(5)	975^c^(4)	1,295^b^(5)	1,683^a^(6)	749^c^(4)
ROH 4–8 Mb	527^b,c^(4)	698^b^(5)	640^b^(3)	1,002^a^(4)	426^c^(4)
ROH > 8 Mb	473^b^(6)	581^b^(7)	459^b^(5)	1,081^a^(7)	576^b^(6)
Homozygosity coverage, %					
ROH 1–2 Mb	83.3	79.6	80.5	70.4	90.5
ROH 2–4 Mb	7.0	8.8	10.6	13.2	4.1
ROH 4–8 Mb	5.1	6.3	5.2	7.9	2.3
ROH > 8 Mb	4.6	5.3	3.7	3.7	3.1

^a–c^Different letters in the column represent significant differences across goat breeds (*P* < 0.05, Tukey test)

*SAA* Saanen, *CAM* Camosciata delle Alpi, *MUR* Murciano-Granadina, *MAL* Maltese, *SAR* Sarda. Standard deviations are reported in parentheses

#### Runs of heterozygosity

**Fig. 3:**
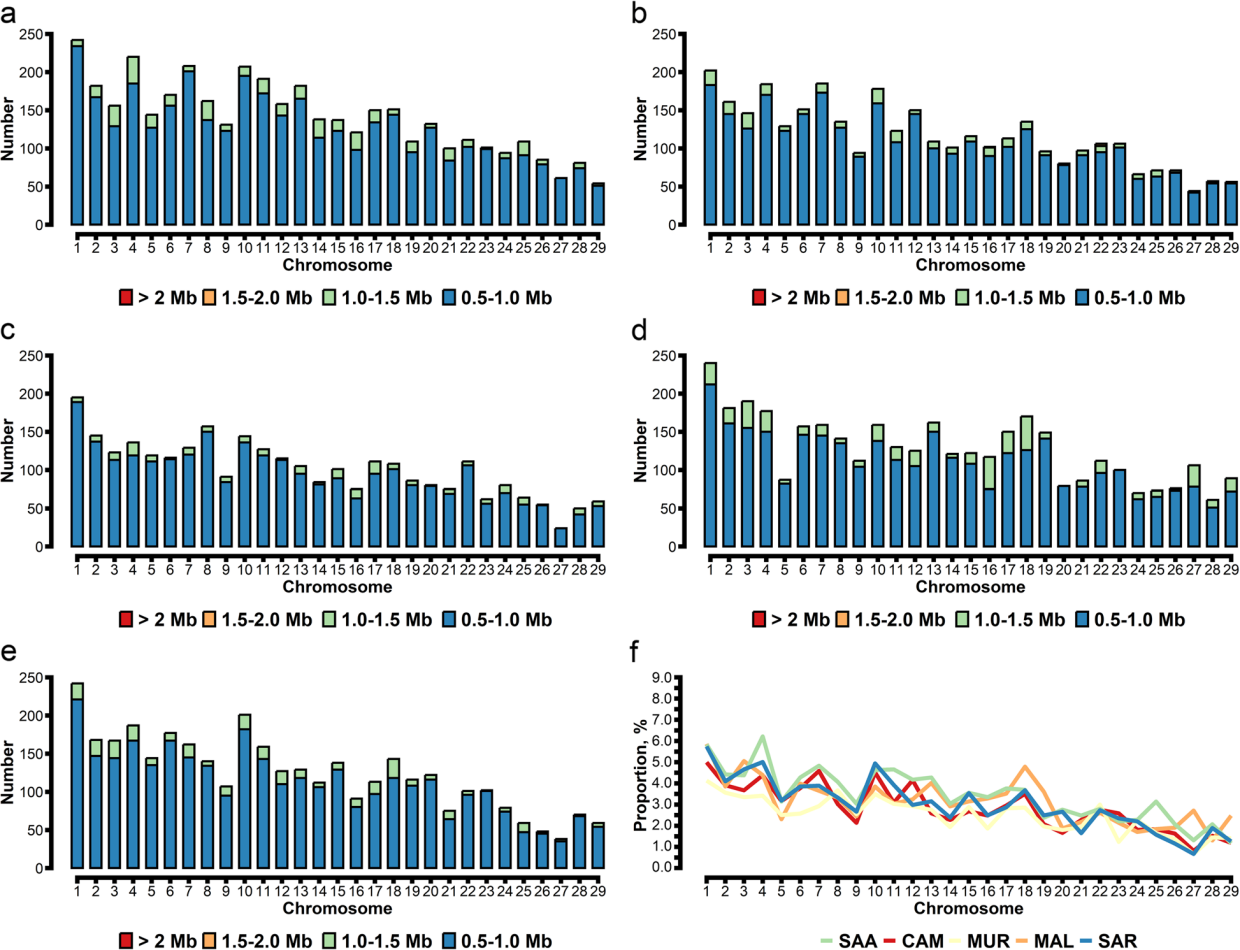
Runs of heterozygosity (ROHet) distribution across the genome for the different goat breeds. Runs of heterozygosity (ROHet) distribution for each chromosome considering different ROHet length classes for different goat breeds. SAA: Saanen (**a**); CAM: Camosciata delle Alpi (**b**); MUR: Murciano-Granadina (**c**); MAL: Maltese (**d**); SAR: Sarda (**e**); Average percentage of autosome chromosome covered by ROHet in goats (**f**)

The heterozygosity coefficient, calculated based on the identified ROHet segments across different goat breeds, is displayed in Fig. S2. The highest heterozygosity coefficient was found for SAA (0.100 ± 0.016), while the other breeds had similar values (Table S3). The ROHet was also counted among the five tested goat breeds. In total, we detected 4,087 ROHet for SAA, 3,360 for CAM, 2,927 for MUR, 3,701 for MAL, and 3,576 for SAR (Table 2). The average number of ROHet segments was 42.57 for SAA, 38.22 for CAM, 33.64 for MUR, 38.55 for MAL, and 31.92 for SAR (Table S3). No ROHet segments > 1.5 Mb were found in the target goat breeds except for four 1.5–2.0 Mb segments in CAM. Regarding ROHet length distribution, we found a higher proportion of 0.5–1.0 Mb segments for all breeds, ranging from 87.49% of homozygosity coverage for MAL to 92.83% for MUR (Table 2). In the specific length category examined, SAA displayed a greater number of segments ranging from 0.5 to 1.0 Mb compared to other breeds (*P* < 0.05), while MUR exhibited a count lower than that of MAL, SAA, and SAR (*P* < 0.05). On the other hand, MAL exhibited the highest count of segments ranging from 1.0 to 1.5 Mb compared to other breeds (*P* < 0.05), while MUR and CAM presented counts lower than those of SAA and SAR (*P* < 0.05). However, when considering the average ROHet length, SAA had the highest value (19.03 Mb), and SAR had the lowest (13.88 Mb) (Table S3). The ROHet distributions across chromosomes are displayed in Fig. 3a–f. The highest number of ROHet was found on CHR 1 for all goat breeds, varying from 242 for SAA and SAR to 195 for MUR, followed by CHR 4 (184) and CHR 7 for CAM (185), CHR 3 for MAL (190), CHR 8 for MUR (157), CHR 4 for SAA (220), and CHR 10 for SAR (201). Unlike the ROH ratios (Fig. 2f), the ROHet ratios of the SAA breed were generally greater than those of the other breeds over the 29 autosomes, except for CHR 3, CHR 18, CHR 27, and CHR 29, where the MAL breed exhibited the highest ROHet ratio, and CHR 10, where the SAR breed had the highest ROHet ratio (Fig. 3f).

**Table 2:** Descriptive statistics of runs of heterozygosity (ROHet) number and coverage across the genome for different length classes in mega basis (Mb)

Item	Alpine breeds		Mediterranean breeds		
	SAA	CAM	MUR	MAL	SAR
Heterozygosity number					
ROHet 0.5–1.0 Mb	3,697 (43.54)^a^	3,109 (37.33)^b^	2,717 (35.25)^c^	3,238 (36.91)^b^	3,246 (43.22)^b^
ROHet 1.0–1.5 Mb	390 (8.04)^b^	251 (5.16)^c^	210 (4.01)^c^	463 (11.54)^a^	330 (6.73)^b^
ROHet 1.5–2.0 Mb	0	4(0.58)	0	0	0
ROHet > 2.0 Mb	0	0	0	0	0
Heterozygosity coverage, %					
ROHet 0.5–1.0 Mb	90.46	92.42	92.83	87.49	90.77
ROHet 1.0–1.5 Mb	9.54	7.46	7.17	12.51	9.23
ROHet 1.5–2.0 Mb	0	0.12	0	0	0
ROHet > 2.0 Mb	0	0	0	0	0

^a–c^Different letters in the column represent significant differences across goat breeds (*P* < 0.05, Tukey test)

*SAA* Saanen, *CAM* Camosciata delle Alpi, *MUR* Murciano-Granadina, *MAL* Maltese, *SAR* Sarda. Standard deviations are reported in parentheses

### Inbreeding coefficients

Table 3 displays the mean estimated inbreeding coefficients using various methodologies. The average total *F*_ROH_ was notably higher in MAL (0.160 ± 0.068) compared to CAM (0.113 ± 0.063), MUR (0.114 ± 0.054), SAA (0.86 ± 0.051), and SAR (0.111 ± 0.063), representing the other breeds. Additionally, MAL exhibited the highest inbreeding coefficients calculated for 1–2 Mb, 2–4 Mb, 4–8 Mb, and > 8 Mb ROH segments compared to the other breeds. Similarly, consistent with the *F*_ROH_ estimates, *F*_G_ and *F*_exH_ in MAL were higher compared to those of the other breeds. The correlations among the inbreeding coefficients are illustrated in Fig. S3. Across all breeds, notably high correlations were noted between *F*_ROH_ total and *F*_exH_, *F*_G_, and *F*_ROH_ > 8 Mb (*r* > 0.85). However, as the segment length decreased, the correlation coefficients between *F*_ROH_ calculated for different segment lengths and *F*_ROH_, *F*_G_, and *F*_exH_, also decreased.

**Table 3:** Descriptive statistics of inbreeding coefficient based on genomic relationship matrix (*F*_G_), runs of homozygosity (ROH) for different lengths, total of ROH (*F*_ROH Total_), and excess of homozygosity (*F*_exH_)

Item	Alpine breeds		Mediterranean breeds		
	SAA	CAM	MUR	MAL	SAR
*F*_G_					
Mean	0.078	0.082	0.099	0.132	0.062
Median	0.074	0.059	0.082	0.122	0.039
Min	0.000	-0.103	0.001	0.009	-0.006
Max	0.344	0.274	0.324	0.296	0.300
SD	0.059	0.064	0.055	0.066	0.060
*F*_ROH Total_					
Mean	0.086	0.113	0.114	0.160	0.111
Median	0.071	0.091	0.099	0.151	0.093
Min	0.029	0.032	0.021	0.036	0.031
Max	0.330	0.316	0.320	0.329	0.323
SD	0.051	0.063	0.054	0.068	0.063
*F*_exH_					
Mean	0.023	0.028	0.030	0.041	0.044
Median	-0.001	-0.003	0.011	0.021	0.022
Min	-0.020	-0.089	-0.038	-0.044	-0.027
Max	0.307	0.228	0.278	0.220	0.295
SD	0.040	0.052	0.047	0.057	0.058
*F*_ROH_ 1–2 Mb					
Mean	0.036	0.042	0.048	0.042	0.060
Median	0.035	0.042	0.050	0.043	0.060
Min	0.024	0.020	0.021	0.022	0.030
Max	0.054	0.053	0.062	0.054	0.096
SD	0.006	0.006	0.007	0.005	0.011
*F*_ROH_ 2–4 Mb					
Mean	0.012	0.016	0.020	0.024	0.010
Median	0.010	0.016	0.020	0.023	0.009
Min	0.002	0.005	0.001	0.004	0.001
Max	0.037	0.041	0.040	0.038	0.034
SD	0.007	0.007	0.006	0.007	0.007
*F*_ROH_ 4–8 Mb					
Mean	0.015	0.021	0.020	0.027	0.014
Median	0.015	0.018	0.020	0.027	0.012
Min	0.002	0.004	0.002	0.005	0.002
Max	0.039	0.064	0.038	0.057	0.044
SD	0.010	0.013	0.009	0.012	0.010
*F*_ROH_ > 8 Mb					
Mean	0.042	0.053	0.042	0.083	0.058
Median	0.026	0.028	0.020	0.069	0.037
Min	0.004	0.004	0.003	0.003	0.003
Max	0.277	0.241	0.266	0.234	0.265
SD	0.047	0.058	0.054	0.064	0.059

*SAA* Saanen, *CAM* Camosciata delle Alpi, *MUR* Murciano-Granadina, *MAL* Maltese, *SAR* Sarda

### Candidate genes and biological pathways in ROH hotspots

**Fig. 4:**
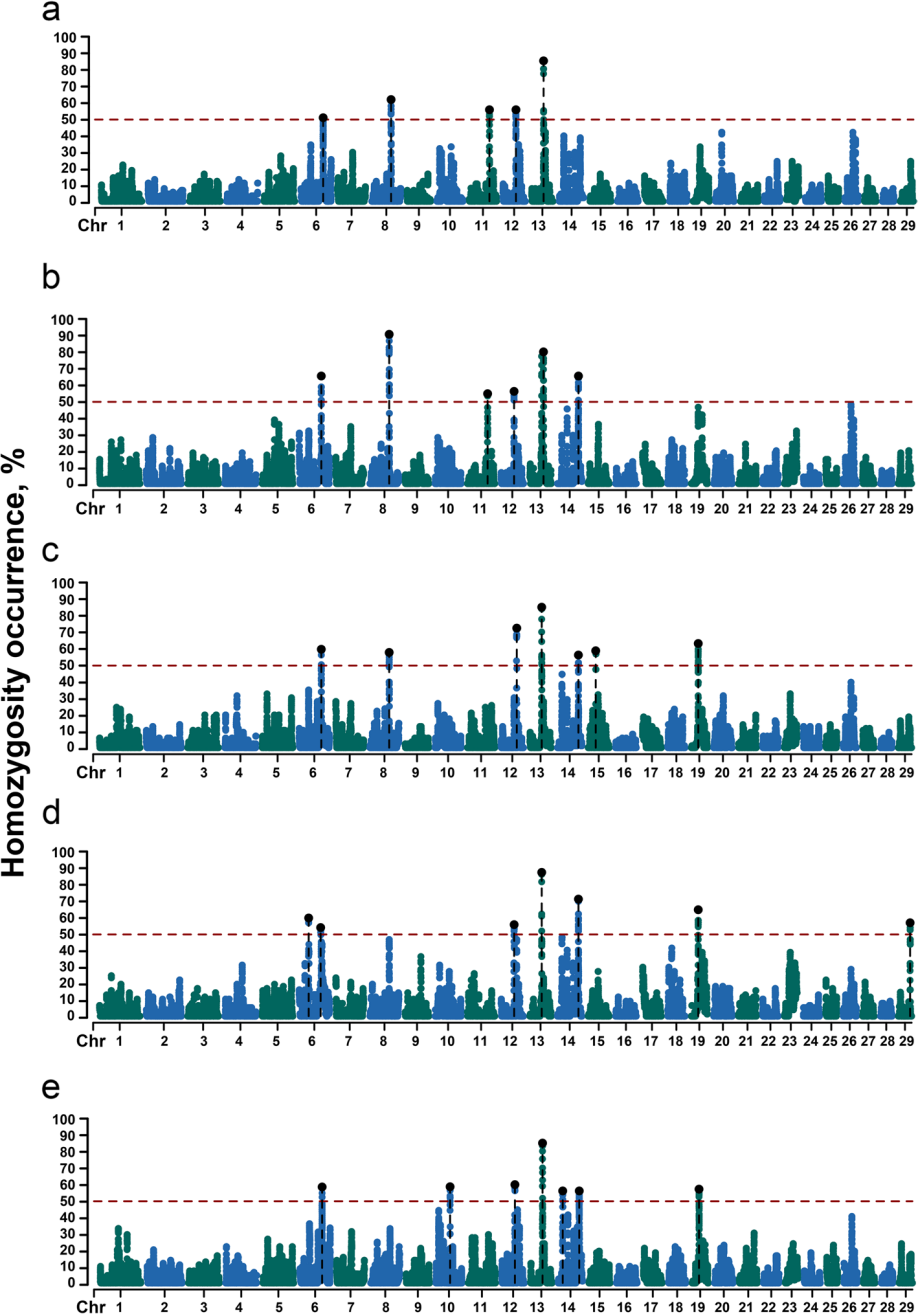
Manhattan plot of the distribution of runs of homozygosity (ROH) islands across the genome for goat breeds. The *X*-axis represents the distribution of ROH across the genome. The *Y*-axis shows the frequency (%) of ROH, and the dashed red line represents the significance level of the top 0.5% of genomic regions SAA: Saanen (**a**); CAM: Camosciata delle Alpi (**b**); MUR: Murciano-Granadina (**c**); MAL: Maltese (**d**); SAR: Sarda (**e**)

The occurrence frequency of SNPs in the identified ROH was counted in 97 SAA, 88 CAM, 87 MUR, 96 MAL, 112 SAR and goats. The regions with 50% of the most frequently occurring SNPs (ROH hotspots) were defined as putative candidate regions under selection (Fig. 4a).

The threshold of SNP occurrence was 50.67% in SAA goats, and five separate ROH hotspots were detected, including 105 SNPs on five autosomes as candidate loci. A total of 26 candidate genes were in these windows (Table 4). The threshold of SNP occurrence was 51.04% in CAM goats, and 11 separate ROH hotspots were detected, including 213 SNPs on six autosomes as candidate loci. A total of 59 candidate genes were mapped in the ROH island windows (Table 4). The threshold of SNP occurrence was 50.59% in MUR goats, and seven separate ROH hotspots were detected, including 200 SNPs on seven autosomes as candidate loci. A total of 77 candidate genes were located within the ROH island windows (Table 4). The threshold of SNP occurrence was 51.27% in MAL goats, and seven separate ROH hotspots were detected, including 209 SNPs on six autosomes as candidate loci. A total of 90 candidate genes mapped in the ROH island windows (Table 4). The threshold of SNP occurrence was 50.00% in SAR goats, including 189 SNPs on six autosomes as candidate loci. A total of 74 candidate genes were in these windows (Table 4).

**Table 4:** Candidate genes of surround runs of homozygosity island in Alpine and Mediterranean goats’ breeds

Chromosome	Position		Autozygosity, %	Gene
	Start	End		
Alpine breeds:				
SAA				
6	86,056,773	86,076,276	51.11	-
8	74,702,203	75,024,344	58.37	*B4GALT1*,* U1*,* SPINK4*,* CHMP5*,* NFX1*,* AQP3*,* NOL6*,* UBE2R2*
11	78,348,517	78,577,786	54.56	*SDC1*,* LAPTM4A*,* MATN3*,* WDR35*
12	50,102,227	50,675,369	53.2	*ATP12A*,* RNF17*,* CENPJ*,* PARP4*,* MPHOSPH8*,* PSPC1*,* ZMYM5*,* ZMYM2*,* GJA3*
13	46,023,812	46,329,866	82.48	*DIP2C*, *U6*, *SNORD31*, *ZMYND11*
CAM				
6	85,882,277	86,076,276	57.49	*SULT1E1*
8	74,508,923	75,367,980	76.63	*APTX*, *SMU1*, *B4GALT1*, *U1*, *SPINK4*, *CHMP5*, *NFX1*, *AQP3*, *NOL6*, *UBE2R2*, *UBAP2*, *SNORD121A*, *DCAF12*, *U6*
11	78,418,240	78,577,786	54.44	*LAPTM4A*, *MATN3*, *WDR35*
12	50,221,647	50,675,369	53.53	*RNF17*, *CENPJ*, *PARP4*, *MPHOSPH8*, *PSPC1*, *ZMYM5*, *ZMYM2*, *GJA3*
13	45,944,824	46,174,021	71.43	*DIPC*, *U6*
13	52,782,768	53,376,104	53.73	*GINS1*, *MYT1*, *NPBWR2*, *OPRL1*, *LKAAEAR1*, *RGS19*, *TCEA2*, *SOX18*, *PRPF6*, *U6*, *SAMD10*, *ZNF512B*, *UCKL1*, *TPD52L2*, *ABHD16B*, *ZBTB46*, *SLC2A4RG*, *ZGPAT*, *ARFRP1*
14	81,319,132	81,451,654	60.60	*HSF1*, *DGAT1*, *SCRT1*, *TMEM249*, *FBXL6*, *SLC52A1*, *ADCK5*, *CPSF1*, *SLC39A4*, *VPS28*, *TONSL*, *ZFTRAF1*
Mediterranean breeds:				
MUR				
6	86,011,549	86,081,135	58.47	-
8	74,663,065	74,934,130	55.32	*B4GALT1*, *U1*, *SPINK4*, *CHMP5*, *NFX1*
12	60,040,786	61,020,592	67.99	*NBEA*, *MAB21L1*
13	45,868,691	46,454,224	80.16	*LARP4B*, *DIP2C*, *U6*, *SNORD31*, *ZMYND11*
14	81,210,832	81,333,756	53.66	*MAF1*, *HGH1*, *MROH1*, *BOP1*, *SCX*, *HSF1*, *DGAT1*
15	29,223,207	30,497,267	58.21	*KCNE3*, *PGM2L1*, *P4HA3*, *PPME1*, *C2CD3*, *UCP3*, *UCP2*, *DNAJB13*, *PAAF1*, *COA4*, *MRPL48*, *RAB6A*, *PLEKHB1*, *FAM168A*, *U6*, *RELT*, *ARHGEF17*, *P2RY6*, *P2RY2*, *FCHSD2*
19	26,662,281	27,074,257	58.53	*ASGR2*, *ASGR1*, *DLG4*, *ACADVL*, *MIR324*, *DVL2*, *PHF23*, *GABARAP*, *CTDNEP1*, *ELP5*, *CLDN7*, *SLC2A4*, *YBX2*, *GPS2*, *NEURL4*, *ACAP1*, *TMEM95*, *TNK1*, *PLSCR3*, *TMEM256*, *NLGN2*, *SPEM1*, *SPEM2*, *TMEM102*, *FGF11*, *CHRNB1*, *ZBTB4*, *POLR2A*, *TNFSF12*, *TNFSF13*, *SENP3*, *EIF4A1*, *SNORA48*, *SNORD10*, *CD68*, *MPDU1*, *SOX15*, *FXR2*
MAL				
6	36,585,519	36,982,281	58.23	*HERC3*, *NAP1L5*, *PYURF*, *HERC5*, *HERC6*, *PPM1K*
6	83,526,335	84,218,404	51.27	*CENPC*, *STAP1*, *UBA6*, *GNRHR*
12	50,554,041	51,186,124	54.36	*ZMYM2*, *GJA3*, *GJB2*, *GJB6*, *CRYL1*, *IFT88*, *IL17D*, *EEF1AKMT1*, *XPO4*, *LATS2*, *SAP18*, *ZDHHC20*
13	45,944,824	46,196,889	85.62	*DIP2C*, *U6*
14	81,297,625	81,478,514	61.36	*BOP1*, *HSF1*, *DGAT1*, *SCRT1*, *TMEM249*, *FBXL6*, *SLC52A1*, *ADCK5*, *CPSF1*, *SLC39A4*, *VPS28*, *TONSL*, *ZFTRAF1*, *KIFC2*, *FOXH1*
19	26,662,281	26,986,682	58.31	*ASGR2*, *ASGR1*, *DLG4*, *ACADVL*, *MIR324*, *DVL2*, *PHF23*, *GABARAP*, *CTDNEP1*, *ELP5*, *CLDN7*, *SLC2A4*, *YBX2*, *GPS2*, *NEURL4*, *ACAP1*, *TMEM95*, *TNK1*, *PLSCR3*, *TMEM256*, *NLGN2*, *SPEM1*, *SPEM2*, *TMEM102*, *FGF11*, *CHRNB1*, *ZBTB4*, *POLR2A|*
29	45,698,809	46,067,919	53.91	*PNPLA2*, *RPLP2*, *SNORA52*, *PIDD1*, *SLC25A22*, *CEND1*, *GATD1*, *EPS8L2*, *TMEM80*, *DEAF1*, *DRD4*, *5S_rRNA*, *CDHR5*, *IRF7*, *PHRF1*, *RASSF7*, *PGGHG*, *IFITM5*, *HRAS*, *RNH1*, *PTDSS2*, *ANO9*, *SIGIRR*
SAR				
6	86,013,561	86,080,969	56.72	-
10	54,961,457	55,210,193	56.84	*VPS13C*, *U6*
12	50,253,456	50,813,808	58.07	*CENPJ*, *PARP4*, *MPHOSPH8*, *PSPC1*, *ZMYM5*, *ZMYM2*, *GJA3*, *GJB2*, *GJB6*, *CRYL1*
13	45,868,691	46,368,408	82.05	*LARP4B*, *DIP2C*, *U6*, *SNORD31*, *ZMYND11*
14	16,887,785	17,689,652	50.05	*OSR2*, *MIR599*, *SNORA70*
14	81,091,465	81,458,794	50.02	*PARP10*, *GRINA*, *SPATC1*, *OPLAH*, *EXOSC4*, *GPAA1*, *CYC1*, *SHARPIN*, *MAF1*, *HGH1*, *MROH1*, *BOP1*, *SCX*, *HSF1*, *DGAT1*, *SCRT1*, *TMEM249*, *FBXL6*, *SLC52A1*, *ADCK5*, *CPSF1*, *SLC39A4*, *VPS28*, *TONSL*, *ZFTRAF1*, *KIFC2*
19	26,610,610	26,907,844	55.82	*RNASEK*, *C17orf49*, *chi-mir-195*, *chi-mir-497*, *BCL6B*, *SLC16A13*, *SLC16A11*, *ASGR2*, *ASGR1*, *DLG4*, *ACADVL*, *MIR324*, *DVL2*, *PHF23*, *GABARAP*, *CTDNEP1*, *ELP5*, *CLDN7*, *SLC2A4*, *YBX2*, *GPS2*, *NEURL4*, *ACAP1*, *TMEM95*, *TNK1*, *PLSCR3*, *TMEM256*, *NLGN2*

*SAA* Saanen, *CAM* Camosciata delle Alpi, *MUR* Murciano-Granadina, *MAL* Maltese, *SAR* Sarda

Functional analyses revealed significant (*q* < 0.1) results only for the SAA breed. Specifically, KEGG analyses showed the presence on the candidate involved in carbohydrate metabolism (*B4GALT1*) belonging to glycosaminoglycan biosynthesis—keratan sulfate (chx00533), glycosphingolipid biosynthesis—lacto and neolacto series (chx00601), galactose metabolism (chx00052) and various types of N-glycan biosynthesis (chx00513) and related to excretory system (*AQP3*) belonging to vasopressin-regulated water reabsorption pathway (chx04962) (Table S4).

**Fig. 5:**
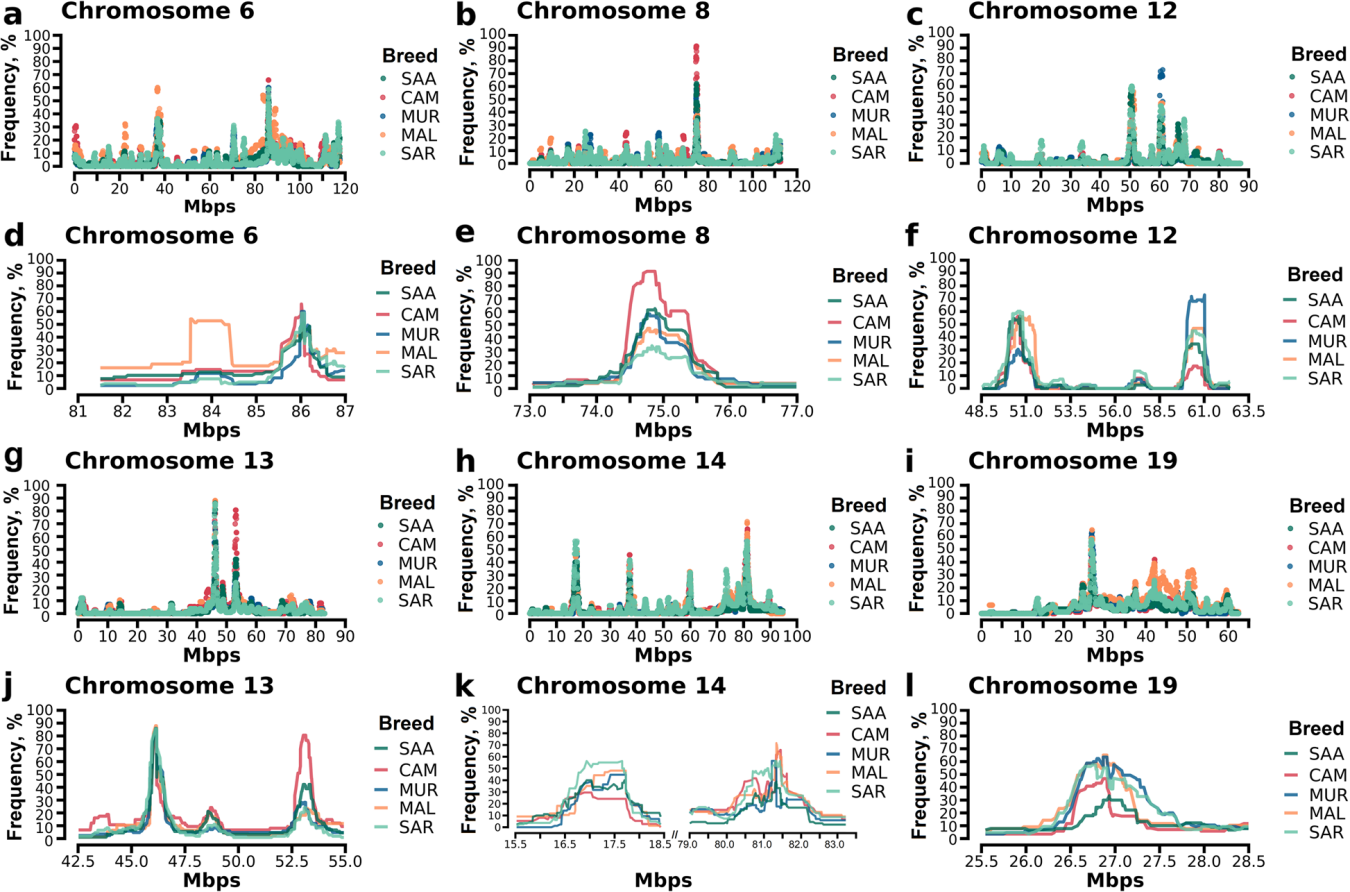
Runs of homozygosity hotspots across goat breeds. Runs of homozygosity hotspots across goat breeds for genomic regions surrounding the ROH regions with a frequency higher than 50%. CHR6 (86.01–86.08 Mb) (**a**, **d**); CHR8 (74.70–74.93 Mb) (**b**, **e**); CHR12 (50.55–50.68 Mb) (**c**, **f**); CHR13 (46.02–46.17 Mb) (**g**, **j**); CHR 14 (81.09–81.33 Mb) (**h**, **k**); CHR 19 (26.66–26.91 Mb) (**i**, **l**). SAA: Saanen; CAM: Camosciata delle Alpi; MUR: Murciano-Granadina; MAL: Maltese; SAR: Sarda

Strikingly, overlapping high-frequency regions of ROH were detected on CHR 6 (86.01–86.08 Mb) for SAA, CAM, MAL, and SAR; on CHR 8 for SAA, CAM and MUR (74.70–74.93 Mb), which included the candidate genes *B4GALT1*,* U1*,* SPINK4*,* CHMP5*,* NFX1*; on CHR 12 (50.55–50.68 Mb) for SAA, CAM, MAL, and SAR, which included the candidate genes *ZMYM2* and *GJA3*; on CHR 13 (46.02–46.17 Mb) for all breeds, which included the candidate genes *DIP2C* and *U6*; on CHR 14 (81.09–81.33) for all breeds except for SAA, which included the candidate genes *HSF1* and *DGAT1*; and on CHR 19 (26.66–26.91 Mb) for MUR, MAL and SAR, which included the candidate genes *ASGR2*, *ASGR1*, *DLG4*, *ACADVL*, *MIR324*, *DVL2*, *PHF23*, *GABARAP*, *CTDNEP1*, *ELP5*, *CLDN7*, *SLC2A4*, *YBX2*, *GPS2*, *NEURL4*, *ACAP1*, *TMEM95*, *TNK1*, *PLSCR3*, *TMEM256*, and *NLGN2* (Table 4, Fig. 5).

### Candidate genes in ROHet hotspots

**Fig. 6:**
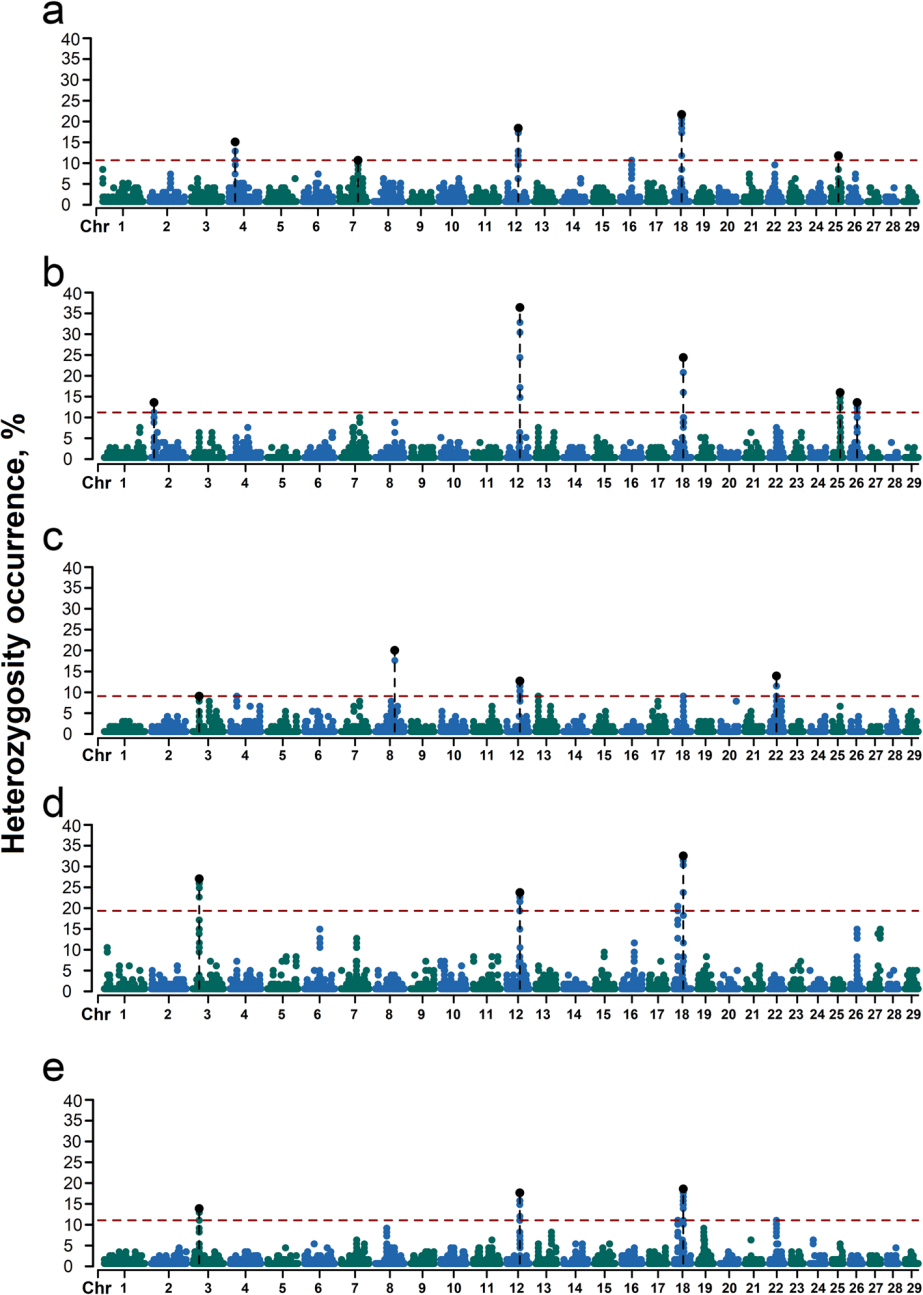
Manhattan plot of the distribution of runs of heterozygosity (ROHet) islands across the genome for goat breeds. The X-axis represents the distribution of ROHet across the genome. The Y-axis shows the frequency (%) of ROHet, and the dashed red line represents the significance level of the top 0.1% of genomic regions. SAA: Saanen (**a**); CAM: Camosciata delle Alpi (**b**); MUR: Murciano-Granadina (**c**); MAL: Maltese (**d**); SAR: Sarda (**e**)

ROHet hotspots were identified by including the top 0.1% of SNPs with occurrence frequencies that correspond to frequency thresholds of 10.08%, 12.22%, 8.65%, 20.16%, and 10.56% in SAA, CAM, MUR, MAL, and SAR goats (Table 5, Fig. 6). Thirty-one candidate genes were revealed on five autosomes in SAA, 35 genes on five autosomes in CAM, 28 genes on four autosomes in MUR, 46 genes on four autosomes in MAL, and 45 genes on four autosomes in SAR. Functional analyses revealed significant (*q* < 0.10) results only for the MUR breed. Specifically, GO terms analyses showed the presence of genes involved in gap junction assembly (GO:0016264; *GJB2* and *GJB6*) and positive regulation of cilium assembly (GO:0045724; *CENPJ* and *IFT88*). KEGG analyses revealed the presence of genes involved in nucleocytoplasmic transport (chx03013; *XPO4* and *SAP18*), hippo signaling pathway—multiple species (chx04392; *LATS2*), pentose and glucuronate interconversions (chx00040; *CRYL1*), base excision repair (chx03410; *PARP4*), and vasopressin-regulated water reabsorption (chx04962; *AQP3*) (Table S4).

**Table 5:** Candidate genes of surround runs of heterozygosity island in goats’ breed

Chromosome	Position		Heterozygosity, %	Gene
	Start	End		
Alpine breeds:				
SAA				
4	26,372,454	26,952,126	13.72	*chi-mir-183*, *MIR96*, *chi-mir-182*, *NRF1*, *SMKR12*, *STRIP2*, *AHCYL2*
7	72,075,266	72,233,284	10.08	*ZNF346*, *U6*, *UIMC1*
12	50,695,369	51,276,130	14.9	*GJB6*, *CRYL1*, *IFT88*, *IL17D*, *EEF1AKMT1*, *XPO4*, *LATS2*, *SAP18*, *ZDHHC20*, *MICU2*
18	36,745,438	37,264,951	18.69	*DUS2*, *NFATC3*, *ESRP2*, *PLA2G15*, *SLC7A6*, *SLC7A6OS*, *PRMT7*, *SMPD3*, *ZFP90*, *CDH3*
25	30,414,940	30,825,735	11.19	*SNORD14*
CAM				
2	10,856,376	11,179,885	13.44	*SESN2*, *MED18*, *PHACTR4*, *U4*, *SNORA73*, *RCC1*, *TRNAU1AP*, *SNORD99*, *SNORA61*, *SNORA44*, *RAB42*, *TAF12*, *GMEB1*
12	50,765,370	51,438,276	26.76	*CRYL1*, *IFT88*, *IL17D*, *EEF1AKMT1*, *XPO4*, *LATS2*, *SAP18*, *ZDHHC20*, *MICU2*, *FGF9*
18	36,745,438	37,205,568	19.22	*DUS2*, *NFATC3*, *U6*, *ESRP2*, *PLA2G15*, *SLC7A6*, *SLC7A6OS*, *PRMT7*, *SMPD3*, *ZFP90*
25	30,315,837	30,798,864	13.78	*SNORD14*
26	28,957,065	29,048,325	12.62	*ARMH3*
Mediterranean breeds:				
MUR				
3	25,023,601	25,023,601	8.65	*FAF1*
8	74,934,130	75,365,691	19.61	*AQP3*, *NOL6*, *UBE2R2*, *UBAP2*, *SNORD121A*, *DCAF12*, *U6*
12	50,152,220	51,252,291	10.8	*RNF17*, *CENPJ*, *PARP4*, *MPHOSPH8*, *PSPC1*, *ZMYM5*, *ZMYM2*, *GJA3*, *GJB2*, *GJB6*, *CRYL1*, *IFT88*, *IL17D*, *EEF1AKMT1*, *XPO4*, *LATS2*, *SAP18*,* ZDHHC20*, *MICU2*
22	28,769,035	29,109,938	13.18	*SHQ1*
MAL				
3	25,006,938	25,478,681	25.37	*FAF1*
12	51,206,124	51,855,723	21.89	*ZDHHC20*, *MICU2*, *FGF9*
18	15,464,429	15,686,036	20.16	*CBFA2T3*, *ACSF3*, *CDH15*, *SLC22A31*
18	36,448,138	37,136,273	30.41	*CTCF*, *CARMIL2*, *ACD*, *PARD6A*, *ENKD1*, *C16orf86*, *GFOD2*, *RANBP10*, *TSNAXIP1*, *CENPT*, *THAP11*, *EDC4*, *NRN1L*, *PSKH1*, *PSMB10*, *LCAT*, *SLC12A4*, *DPEP3*, *DPEP2*, *DDX28*, *DUS2*, *NFATC3*, *U6*, *ESRP2*, *PLA2G15*, *SLC7A6*, *SLC7A6OS*, *PRMT7*, *SMPD3*
SAR				
3	24,950,427	25,476,546	12.67	*DMRTA2*, *FAF1*
12	50,302,929	50,937,884	15.32	*IFT88*, *IL17D*,* EEF1AKMT1*,* XPO4*, *LATS2*, *SAP18*, *ZDHHC20*, *MICU2*, *FGF9*
18	15,517,488	15,664,164	10.56	*CBFA2T3*, *ACSF3*, *CDH15*
18	36,448,138	37,136,273	16.67	*CTCF*, *CARMIL2*, *ACD*, *PARD6A*, *ENKD1*, *C16orf86*, *GFOD2*, *RANBP10*, *TSNAXIP1*, *CENPT*, *THAP11*, *EDC4*, *NRN1L*, *PSKH1*, *PSMB10*, *LCAT*, *SLC12A4*, *DPEP3*, *DPEP2*, *DDX28*, *DUS2*, *NFATC3*, *U6*, *ESRP2*, *PLA2G15*, *SLC7A6*, *SLC7A6OS*, *PRMT7*, *SMPD3*

*SAA* Saanen, *CAM* Camosciata delle Alpi, *MUR* Murciano-Granadina, *MAL* Maltese, *SAR* Sarda

**Fig. 7:**
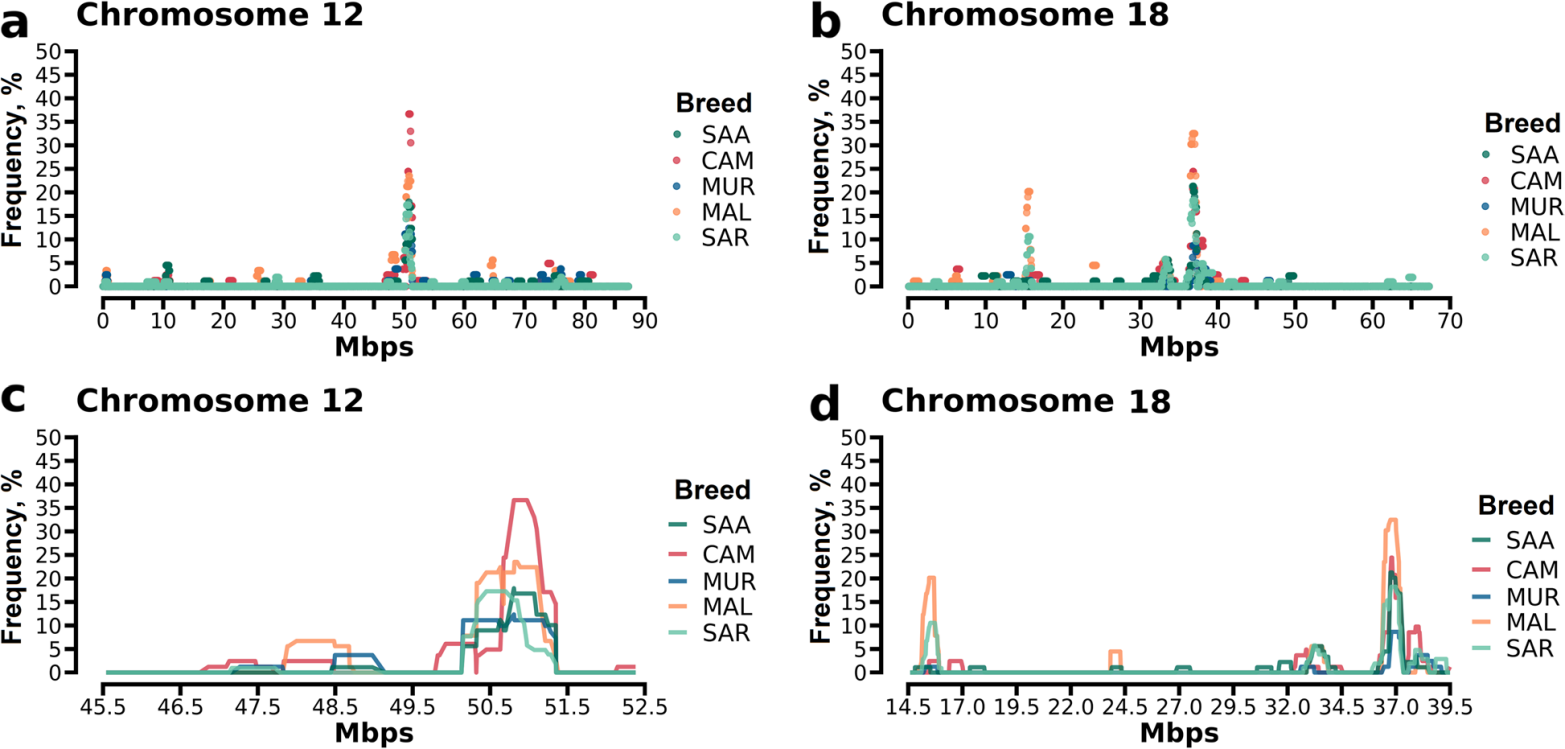
Runs of heterozygosity hotspots shared across goat breeds. Runs of heterozygosity hotspots shared across goat breeds for genomic regions considering the threshold of top 0.1%. CHR12 (50.76–50.94 Mb) (**a**, **c**); CHR18 (36.75–37-14 Mb) (**b**, **d**). SAA: Saanen; CAM: Camosciata delle Alpi; MUR: Murciano-Granadina; MAL: Maltese; SAR: Sarda

Universal high-frequency regions of ROHet were detected on CHR 12 (50.76–50.94 Mb), which included the candidate genes *IFT88, IL17D, EEF1, AKMT1, XPO4, LATS2, SAP18, ZDHHC20,* and *MICU2*; and on CHR 18 (36.75–37.14 Mb) except for MUR, which included the candidate genes *DUS2*, *NFATC3*, *U6*, *ESRP2*, *PLA2G15*, *SLC7A6*, *SLC7A6OS*, *PRMT7*, and *SMPD3* (Table 5, Fig. 7).

## Discussion

### Pattern of homozygosity and heterozygosity in goat populations

Runs of homozygosity and ROHet offer insights into the effects of adaptive evolution across the genome. Exploring ROH and ROHet profiles across populations allows to discern differences in demographic history and selective pressures, offering a window into the underlying stochastic processes driving genome-wide diversity [36].

The average number of ROH per animal ranged from 107.72 in SAA to 164.65 in SAR, which are higher than the values reported in Italian goat populations, including MAL (57.1), SAA (25.8), SAR (18.1), and CAM (31.5) [12] sampled in Sardinia, and MAL (38.8) and Girgentana (37.1) sampled in Sicily [37]. It is worth noting, however, that the population size in that study was much lower than that of the present study. Larger sample sizes provide more statistical power and accuracy in detecting ROH, contributing to a better understanding of population genetics and evolutionary processes. The average number of ROHet detected per animal varied across breeds, ranging from 31.93 in SAR to 42.13 in SAA. These values are lower than those reported by Li et al. [38] in Chinese goat populations (45.50) and higher than those reported by Tsartsianidou et al. [33] in Mediterranean domestic Greek sheep (28.28). Nevertheless, our results were consistent with previous studies where the short segments of ROH and ROHet showed the largest proportion and ROHet were much rarer and shorter than ROH [35, 36]. Interestingly, the local SAR breed had the highest ROH number and the highest ROH ratio over the 29 chromosomes, which suggested that it might have experienced a more pronounced level of genetic isolation, or selective pressures compared to other breeds. On the contrary, although SAR had the highest total count of ROHet, the MAL breed exhibited the highest number of longer ROHet, which may explain why the coverage rates were not always greater in SAR on the 29 autosomes. The average number and length of ROH were significantly higher than those of ROHet in the tested populations (ANOVA, *P* < 0.01). Also, the incidence of SNP in ROH was significantly higher than that in ROHet (ANOVA, *P* < 0.0015). As expected, Pearson correlation between ROH and ROHet varied across breeds with values of −0.38 for CAM (*P* = 0.0382), −0.58 for MAL (*P* = 2.301e-05), −0.41 for MUR (*P* = 0.0187), −0.37 for SAA (*P* = 0.039) and −0.50 for SAR (*P* = 0.00026).

### Inbreeding level in goat populations

The inbreeding coefficients across different populations could reflect the size of population, the breeding practices and historical management of these breeds. The inbreeding coefficient estimated using ROH was larger than what reported in previous studies on Italian goat populations [12, 37]. Moderate *F*_ROH_ values were detected for all sampled breeds (0.10 < *F*_ROH_ < 0.20), which could be reflective of controlled breeding strategies aimed at maintaining specific traits. MAL breed has higher inbreeding coefficients, which suggests the need to implement strategies to mitigate inbreeding and preserve genetic diversity within the population. On the other hand, SAA breed showed the lowest values (*F*_ROH_ < 0.10), suggesting that this breed has higher genetic diversity due to diverse breeding practices or a larger effective population size, which is essential for the long-term sustainability of the breed. Correlations between *F*_*ROH*_ total and *F*_exH_, *F*_G_, and *F*_ROH_ > 8 Mb were high, while *F*_ROH_ 1-2 Mb displayed the lowest correlations with the other inbreeding coefficients, in line with previous studies on other species [14, 39, 40] including goat [38, 41]. Short ROH primarily stems from ancient inbreeding episodes [42] and may not represent the complete autozygosity of the sample. Additionally, some of these homozygous stretches may be identical by state rather than by descent, potentially resulting from low recombination rates or high linkage disequilibrium in unrelated ancestors [43].

### Candidate genes in ROH hotspots

Typically, both natural and artificial selection lead to a rise in ROH frequency [44], and regions with high ROH concentration are also subject to positive selection [45]. In the SAA population, we found genes in ROH hotspots that were enriched in KEGG pathways related to carbohydrate metabolism. In particular, *B4GALT1* is an important candidate gene for milk performance traits in cattle and encodes the catalytic part of lactose synthesis [46]. This gene was also included in the ROH hotspot on CHR 8 which was shared among SAA, CAM and MUR breeds. Furthermore, *AQP3,* which belong to the hypoxia-related genes*,* not only has been associated with immunity and proposed as a candidate gene for selection signature in Brazilian sheep breeds [47]. Additionally, it has been identified as a regulator of thermal adaptation, due to its role in controlling evapotranspiration and facilitating cryoprotectant transport [48]. These same genes were also identified as potentially undergoing selection according to the findings of Li et al. [38] in Chinese goat breeds. Bertolini et al. [6] detected a shared ROH region on CHR 12 (50–51 Mb) in global goat breeds. In this study, we confirmed the presence of a family of sensory organ genes, including *GJA3* in all breeds except for MUR, and also *GJB2* and *GJB6 i*n MAL and SAR as also shown by Li et al. [38]. Variants in *GJA3* have been associated with cataracts in humans [49], while mutations in *GJB2* and *GJB6,* encoding for connexin-26 and 30, respectively, transmembrane proteins involved in the formation of gap junction in the cochlea, are known for their critical roles in hearing [50]. This functional region is located in a common ROH hotspot among different worldwide goat breeds, and these genes were selected positively [38, 51, 52]. The genomic region associated with perception may have undergone selection even prior to the domestication of goats, which would have benefited from enhanced senses of sight and hearing to forage and detect potential threats in their natural habitats. *ZMYM2* is related to the regulation of spermatogenesis and cell cycle in goats [53] and seems to be implicated in adaptive processes in Mediterranean sheep and goats [54]. An interesting candidate gene identified exclusively in the MUR breed is *NBEA*, which encodes neurobeachin, a brain-specific A-kinase anchor protein essential for the synaptic surface expression of glutamate and GABA receptors. It is believed to play a critical role in thermal adaptation by regulating synaptic transmission [55]. Indeed, this gene has been correlated with environmental gradients by Serranito et al. [54]. Additionally, *NBEA* has been associated with high altitude in both Ethiopian [56] and Chinese sheep [57], and with body temperature regulation in cattle [58]. Regarding genes related to environmental adaptation, although the sampled breeds originate from different countries and environments (e.g., alpine versus Mediterranean), they are all of European origin. Consequently, the limited genetic differences observed in the ROH hotspots were largely expected. Indeed, it has been recently evidenced that, after domestication, genetic signature among European goat breeds have been mitigated by the intense migratory movements facilitated by the absence of geographic obstacles (e.g. deserts), if compared to Asian and African ones [59].

Concerning the ROH hotspot on CHR13 shared among all breeds, this region mapped two genes, i.e., *DIP2C* and *U6*. While the biological functions of *DIP2C* are not entirely clear, it may regulate neuronal differentiation and early embryonic nervous development [60]. This gene was associated with physiological and anatomical indicators of heat stress response in lactating sows [61]. *U6*, which encodes for a small nuclear RNA that plays a role in RNA splicing, was among the genes under selection that affected milk production traits in sheep [62]. On the ROH hotspot on CHR 14, which is shared among all breeds except for SAA, we encountered *HSF1* and *DGAT1*. *HSF1,* known as the primary regulator of the heat shock transcriptional response, also plays significant roles in mammalian embryonic development and gametogenesis [63]. This gene was proposed as candidate gene in cattle and goat [64, 65]. The key regulator of milk fat production *DGAT1* which catalyzes the last step in triglyceride (TAG) synthesis: the esterification of a fatty acyl-CoA to the *sn*-3 position of a diacylglycerol. This gene is found in regions of the genome with a high frequency of ROH occurrence in cattle [64, 66] but it remained undetected in goats so far [43, 67]. Several genes were mapped in the ROH hotspot on CHR 19, which was shared among MAL, MUR and SAR breeds. A *Capra hircus* chromosome 19 locus within *ASGR2* linked to milk production influences mammary conformation [68]. The homologous bovine gene is also located on chromosome 19 and is involved in the regulation of protein stability and lipid homeostasis. Given that udder tissue primarily consists of fat deposition and connective tissue, which includes fibroblasts and adipocytes, both these components play crucial roles in udder formation. On CHR 19, ROH also harbored genes related to immunity (*GABARAP*, *GPS2*) and reproduction (*TMEM95*) as reported by Sallam et al. [69] in Egyptian goat breeds. *GABARAP* is part of a highly conserved gene family with a crucial role in autophagy. Extensive evidence indicates an interaction between autophagy, apoptosis, and the immune response [70]. *GPS2* is a versatile protein involved in regulating inflammation and metabolism in adipose tissue, the liver, and immune cells [71]. Finally, *TMEM95*, is necessary for sperm-egg interaction and, therefore, plays a key role in mediating the reproduction process [72].

### Candidate genes in ROHet hotspots

In livestock, there is significantly less understanding of ROHet hotspots compared to ROH hotspots [14]. However, despite their smaller representation respect to ROH, they have been strongly associated with animal fitness and survival, and heterotic balancing selection processes [13].

Bertolini et al. [6] found two ROH regions on goat CHR 12, from 43 to 44 Mb in European breeds, and from 50 to 51 Mb in worldwide goats, which overlapped the ROHet hotspot on CHR 12 in CAM, MUR, SAA and SAR (50.76–50.94 Mb), harboring genes that are related to immune system (*IFT88*, *IL17D*) as well as reproduction (*LATS2*, *FGF9*, *SAP18*, *MICU2*, *SAP18*). The *IL17D* gene, spanning from ~ 50.90 to ~ 50.93 Mb and part of the IL17 family of cytokines, is connected with host defense and immune response in humans [73]. This region is the same as the one found in five commercial and local goat breeds by Biscarini et al. [74] and by Chessari et al. [17]. Genes, such as *LATS2*, *FGF9*, *SAP18*, *MICU2,* involved in reproduction exhibit high expression levels in human reproductive organs which suggest they could have crucial roles in reproductive processes of both sheep and humans [75]. The second most common ROHet island was on CHR 18 (36.75–37.14 Mb). This region was primarily observed in Italian and Alpines breeds and corresponded to a ROH island identified in goats from Europe, Africa and Asia by Bertolini et al. [6] but also coincides with a ROHet hotspot found by Chessari et al. [17] in worldwide goat populations. Runs of homozygosity and ROHet can both map to the same genes, reflecting different inheritance patterns in the genome. A study on Mediterranean domestic sheep found specific genomic regions where both ROH and ROHet were present, impacting traits related to local adaptation and climate resilience. This overlap of ROH and ROHet might suggest a nuanced genetic structure where both homozygosity and heterozygosity play crucial roles in the adaptation of these animals​ [33]. Among the genes overlapping this region, we found the splicing regulator *ESRP1* which coordinates an epithelial splicing program essential for mammalian development [76]. Genes involved in immune system regulation were also annotated such as the *NFATC2* transcription factor which plays a crucial role in suppression of CD4^+^ T lymphocytes by CD4^+^ CD25^+^ regulatory T cells [77]. *PLA2G15* may also have a role in host defense and in the processing of lipid antigens for presentation by CD1 proteins [78].

## Conclusions

In this study, we provided insights into the genetic diversity within and between five Alpine and Mediterranean goat breeds. By mapping the genomic regions associated with ROH and ROHet, we identified loci potentially under selection pressure. This analysis revealed genes that are important for traits such as milk production (e.g., *DGAT1*,* B4GALT1*), immunity (e.g., *GABARAP*, *GPS2*) and adaptation to harsh environments (e.g., *GJA3*, *GJB2* and *GJB6*), all of which are critical for the sustainability of these breeds in Alpine and Mediterranean regions. High levels of ROH can indicate inbreeding and a lack of genetic variation, which can make populations more susceptible to diseases and reduce their ability to adapt to environmental changes. Conversely, areas with high ROHet suggest regions of the genome where genetic diversity is maintained, which can be beneficial for the health and resilience of the breed. Lastly, this research has practical implications for breeding programs. By understanding the genomic landscape, breeders can make more informed decisions to manage inbreeding and maintain genetic diversity. A limitation of this study, however, is that while our findings offer valuable insights at the gene level, pinpointing the causal variants of the inferred selection signals would require deeper genomic approaches, such as whole-genome sequencing or fine-mapping.

## Supplementary Information

Additional file 1: Fig. S1. Localization of sampled goats’ farms according to the breeds. Saanen (SAA), Camosciata delle Alpi (CAM), Murciano-Granadina (MUR), Maltese (MAL) and Sarda (SAR). Maps of Europe and Sardinia created at Google maps (https://www.google.com/maps).

Additional file 2: Table S1. Sample size after genotyping quality control (N), expected heterozygosity (He), observed heterozygosity (Ho), and proportion of polymorphic SNPs (PN).

Additional file 3: Table S2. Assessment of runs of homozygosity (ROH) and heterozygosity (ROHet) in goat breeds.

Additional file 4: Fig S2. Boxplot of the heterozygosity coefficient for the different goat breeds. Heterozygosity coefficient calculated by identified runs of heterozygosity (ROHet) considering the total segments for different goat breeds. SAA: Saanen; CAM: Camosciata delle Alpi; MUR: Murciano-Granadina; MAL: Maltese; SAR: Sarda.

Additional file 5: Table S3. Descriptive statistics for heterozygosity coefficient calculated by identified runs of heterozygosity (ROHet) for different goat breeds.

Additional file 6: Fig. S3. Correlations among genomic inbreeding coefficients. Scatterplots (lower panel) and Pearson’s correlations (upper panel) of the genomic inbreeding coefficients based on runs of homozygosity (*F*_ROH_) (*F*_ROH Total_, *F*_ROH_ 1–2 Mb, *F*_ROH_ 2–4 Mb, *F*_ROH_ 4–8 Mb, *F*_ROH_ > 8 Mb), and inbreeding coefficient based on genomic relationship matrix (*F*_G_) and inbreeding coefficient based excess of homozygosity (*F*_exH_). SAA: Saanen; CAM: Camosciata delle Alpi; MUR: Murciano-Granadina; MAL: Maltese; SAR: Sarda.

Additional file 7: Table S4. Gene Ontology (GO) and Kyoto Encyclopedia of Genes and Genomes (KEGG) enriched pathways for the candidate genes in the runs of homozygosity (ROH) and heterozygosity (ROHet) hotspots for the different goat breeds.

## Data Availability

Data are available upon request to the corresponding author.

## Abbreviations

ACADVL: Acyl-CoA dehydrogenase very long chain
ACAP1: ArfGAP with coiled-coil, ankyrin repeat and PH domains 1
AQP3: Aquaporin 3
ASGR1: Asialoglycoprotein receptor 1
ASGR2: Asialoglycoprotein receptor 2
B4GALT1: Beta-1, 4 galactosyltransferase 1
CAM: Camosciata delle Alpi
CHMP5: Charged multivesicular body protein 5
CLDN7: Claudin 7
CRYL1: Crystallin lambda 1
CTDNEP1: CTD nuclear envelope phosphatase 1
DGAT1: Diacylglycerol O-acyltransferase 1
DIP2C: Disco interacting protein 2 homolog C
DLG4: Discs large MAGUK scaffold protein 4
DVL2: Dishevelled segment polarity protein 2
EEF1: Eukaryotic translation elongation factor 1 alpha
ELP5: Elongator acetyltransferase complex subunit 5
ESRP1: Epithelial splicing regulatory protein 1
ESRP2: Epithelial splicing regulatory protein 2
GABARAP: GABA type A receptor-associated protein
GJA3: Gap junction protein alpha 3
GJB2: Gap junction protein beta 2
GJB6: Gap junction protein beta 6
GO: Gene Ontology
HSF1: Heat shock transcription factor 1
IFT88: Intraflagellar transport 88
IL17D: Interleukin 17D
KEGG: Kyoto Encyclopedia of Genes and Genomes
LATS2: Large tumor suppressor kinase 2
MAF: Minor allele frequency
MAL: Maltese
MICU2: Mitochondrial calcium uptake 2
MIR324: MicroRNA 324
MUR: Murciano-Granadina
NEURL4: Neuralized E3, ubiquitin protein ligase 4
NFATC2: Nuclear factor of activated T cells 2
NFX1: Nuclear transcription factor, x-box binding 1
NLGN2: Neuroligin 2
PARP4: Poly (ADP-Ribose) polymerase family member 4
PHF23: PHD finger protein 23
PLA2G15: Phospholipase A2 group XV
PLSCR3: Phospholipid scramblase 3
PRMT7: Protein arginine methyltransferase 7
QC: Quality control
ROHet: Runs of heterozygosity
SAA: Saneen
SAP18: Sin3A associated protein 18
SAR: Sarda
SLC2A4: Solute carrier family 2 member 4
SLC7A6: Solute carrier family 7 member 6
SLC7A6OS: Solute carrier family 7 member 6 opposite strand
SMPD3: Sphingomyelin phosphodiesterase 3
SNP: Single nucleotide polymorphism
TMEM256: Transmembrane protein 256
TMEM95: Transmembrane protein 95
TNK1: Tyrosine kinase non receptor 1
U1: U6 small nuclear 1
U6: RNA X-Box binding 1
XPO4: Exportin 4
YBX2: Y-Box binding protein 2
ZDHHC20: Zinc finger DHHC-type palmitoyltransferase 20
ZMYM2: Zinc finger MYM-type containing 2, 4-galactosyltransferase 1
SPINK4: Serine peptidase inhibitor kazal type 4
