# Supplementary figures and images for: Genome-wide landscape of genetic diversity, runs of homozygosity, and runs of heterozygosity in five Alpine and Mediterranean goat breeds
Source: J Anim Sci Biotechnol. 2025 Mar 3;16:33. doi: 10.1186/s40104-025-01155-3 (PMC11874128; doi:10.1186/s40104-025-01155-3)

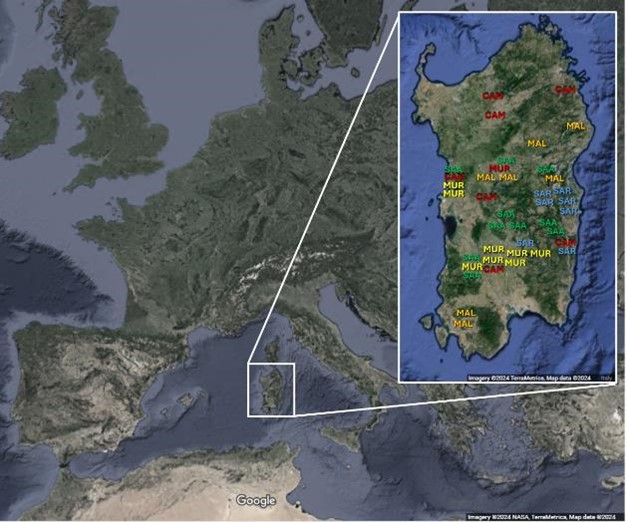

Supplement: Supplementary file 1 — Additional file 1: Fig. S1. Localization of sampled goats’ farms according to the breeds. Saanen (SAA), Camosciata delle Alpi (CAM), Murciano-Granadina (MUR), Maltese (MAL) and Sarda (SAR). Maps of Europe and Sardinia created at Google maps (https://www.google.com/maps). [file 40104_2025_1155_MOESM1_ESM.jpg]

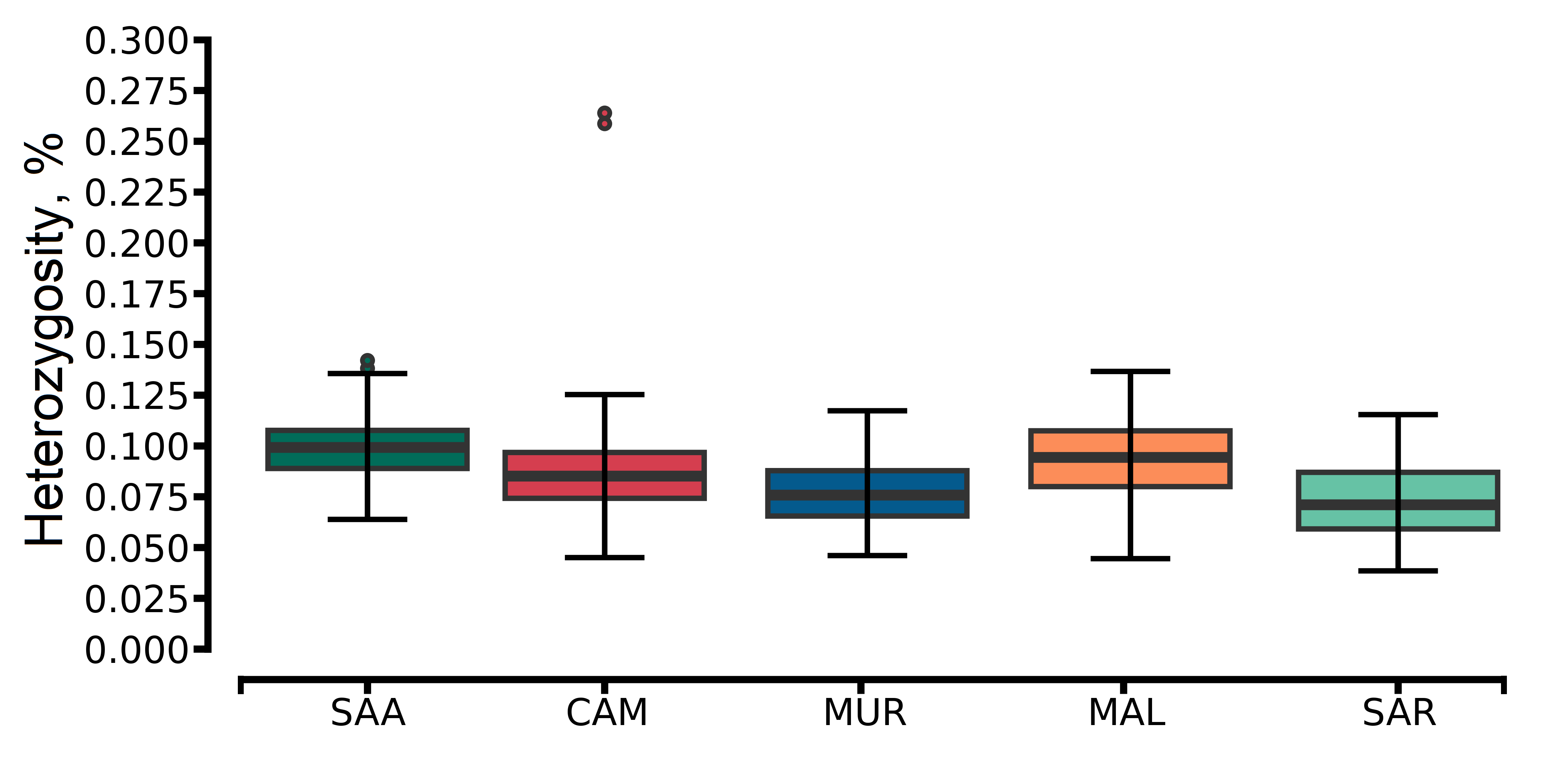

Supplement: Supplementary file 4 — Additional file 4: Fig S2. Boxplot of the heterozygosity coefficient for the different goat breeds. Heterozygosity coefficient calculated by identified runs of heterozygosity (ROHet) considering the total segments for different goat breeds. SAA: Saanen; CAM: Camosciata delle Alpi; MUR: Murciano-Granadina; MAL: Maltese; SAR: Sarda. [file 40104_2025_1155_MOESM4_ESM.tiff]

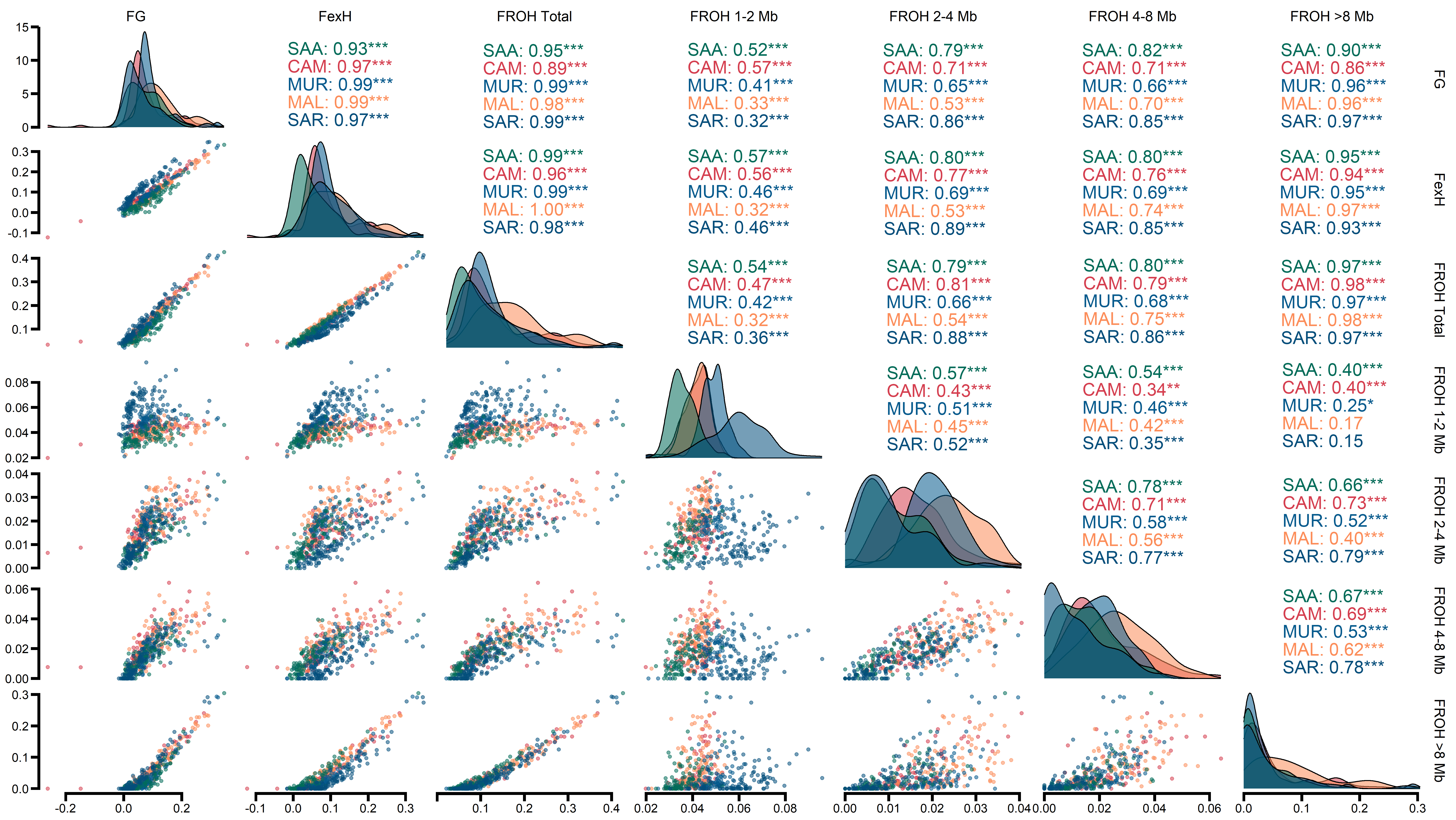

Supplement: Supplementary file 6 — Additional file 6: Fig. S3. Correlations among genomic inbreeding coefficients. Scatterplots (lower panel) and Pearson’s correlations (upper panel) of the genomic inbreeding coefficients based on runs of homozygosity (FROH) (FROH Total, FROH 1–2 Mb, FROH 2–4 Mb, FROH 4–8 Mb, FROH > 8 Mb), and inbreeding coefficient based on genomic relationship matrix (FG) and inbreeding coefficient based excess of homozygosity (FexH). SAA: Saanen; CAM: Camosciata delle Alpi; MUR: Murciano-Granadina; MAL: Maltese; SAR: Sarda. [file 40104_2025_1155_MOESM6_ESM.tiff]
