# Supplementary material for: Genome-wide landscape of genetic diversity, runs of homozygosity, and runs of heterozygosity in five Alpine and Mediterranean goat breeds
Source: J Anim Sci Biotechnol. 2025 Mar 3;16:33. doi: 10.1186/s40104-025-01155-3 (PMC11874128; doi:10.1186/s40104-025-01155-3)
Supplement: Supplementary file 2 — Additional file 2: Table S1. Sample size after genotyping quality control (N), expected heterozygosity (He), observed heterozygosity (Ho), and proportion of polymorphic SNPs (PN). [file 40104_2025_1155_MOESM2_ESM.docx]

# **Supplementary Information**

**Supplementary Table S1.** Sample size after genotyping quality control (N), expected heterozygosity (H_e_), observed heterozygosity (H_o_), and proportion of polymorphic SNPs (P_N_).

|  | Alpine breeds: | |  | Mediterranean breeds: | | |
| --- | --- | --- | --- | --- | --- | --- |
| Breed | SAA | CAM |  | MUR | MAL | SAR |
| Goats, N | 97 | 88 |  | 87 | 96 | 112 |
| He | 0.40 (0.009) | 0.39 (0.007) | | 0.39 (0.005) | 0.38 (0.006) | 0.39 (0.007) |
| Ho | 0.39 (0.023) | 0.38 (0.018) | | 0.38 (0.025) | 0.37 (0.029) | 0.37 (0.028) |
| P-value | 0.59 | 0.62 |  | 0.62 | 0.60 | 0.59 |
| PN (%) | 99.66  (1.22) | 99.53  (1.22) | | 99.75  (1.16) | 99.45  (1.16) | 99.77  (1.02) |

Standard deviations are reported in parentheses

SAA: Saanen; CAM: Camosciata delle Alpi; MUR: Murciano-Granadina; MAL: Maltese; SAR: Sarda
