# Supplementary material for: Genome-wide landscape of genetic diversity, runs of homozygosity, and runs of heterozygosity in five Alpine and Mediterranean goat breeds
Source: J Anim Sci Biotechnol. 2025 Mar 3;16:33. doi: 10.1186/s40104-025-01155-3 (PMC11874128; doi:10.1186/s40104-025-01155-3)
Supplement: Supplementary file 3 — Additional file 3: Table S2. Assessment of runs of homozygosity (ROH) and heterozygosity (ROHet) in goat breeds. [file 40104_2025_1155_MOESM3_ESM.docx]

**Supplementary Table S2** - Assessment of runs of homozygosity (ROH) and heterozygosity (ROHet) in goat breeds.

| Breed | n | nROH | SD nROH | ROH (Mb) | nROHet | SD nROHet | ROHet (Mb) |
| --- | --- | --- | --- | --- | --- | --- | --- |
| SAA | 96 | 107.72 | 16.752 | 213.115 | 42.57 | 5.458 | 19.03 |
| CAM | 88 | 125.72 | 16.868 | 280.168 | 38.22 | 9.365 | 17.03 |
| MUR | 87 | 140.8 | 18.153 | 282.155 | 33.64 | 4.692 | 14.51 |
| MAL | 96 | 132.34 | 16.388 | 295.702 | 38.55 | 5.735 | 18.02 |
| SAR | 112 | 164.65 | 29.432 | 314.664 | 31.92 | 5.421 | 13.88 |

SAA: Saanen; CAM: Camosciata delle alpi; MUR: Murciano-Granadina; MAL: Maltese; SAR: Sarda.
