# Supplementary material for: Genome-wide landscape of genetic diversity, runs of homozygosity, and runs of heterozygosity in five Alpine and Mediterranean goat breeds
Source: J Anim Sci Biotechnol. 2025 Mar 3;16:33. doi: 10.1186/s40104-025-01155-3 (PMC11874128; doi:10.1186/s40104-025-01155-3)
Supplement: Supplementary file 5 — Additional file 5: Table S3. Descriptive statistics for heterozygosity coefficient calculated by identified runs of heterozygosity (ROHet) for different goat breeds. [file 40104_2025_1155_MOESM5_ESM.docx]

**Supplementary Table S3** – Descriptive statistic for heterozygosity coefficient calculated by identified runs of heterozygosity (ROHet) for different goat breeds.

| Breed | mean | sd | Min | Max |
| --- | --- | --- | --- | --- |
| SAA | 0.100 | 0.016 | 0.06 | 0.14 |
| CAM | 0.089 | 0.029 | 0.05 | 0.26 |
| MUR | 0.077 | 0.014 | 0.05 | 0.13 |
| MAL | 0.092 | 0.018 | 0.04 | 0.13 |
| SAR | 0.074 | 0.017 | 0.04 | 0.12 |

SAA: Saanen; CAM: Camosciata delle alpi; MUR: Murciano-Granadina; MAL: Maltese; SAR: Sarda
